# Transcriptomics for Clinical and Experimental Biology Research: Hang on a Seq

**DOI:** 10.1002/ggn2.202200024

**Published:** 2023-01-17

**Authors:** Tanner Stokes, Haoning Howard Cen, Philipp Kapranov, Iain J Gallagher, Andrew A. Pitsillides, Claude‐Henry Volmar, William E Kraus, James D. Johnson, Stuart M. Phillips, Claes Wahlestedt, James A. Timmons

**Affiliations:** ^1^ Faculty of Science McMaster University Hamilton L8S 4L8 Canada; ^2^ Life Sciences Institute University of British Columbia Vancouver V6T 1Z3 Canada; ^3^ School of Medicine Huaqiao University Xiamen 362021 China; ^4^ School of Applied Sciences Edinburgh Napier University Edinburgh EH11 4BN UK; ^5^ Comparative Biomedical Sciences Royal Veterinary College London NW1 0TU UK; ^6^ Miller School of Medicine University of Miami Miami FL 33136 USA; ^7^ School of Medicine Duke University Durham NC 27701 USA; ^8^ William Harvey Research Institute Queen Mary University London London EC1M 6BQ UK; ^9^ Augur Precision Medicine LTD Stirling FK9 5NF UK

**Keywords:** arrays, cDNA, cRNA, diagnostics, drug repurposing, lncRNA, noncoding RNA, RNA, sequencing, splicing

## Abstract

Sequencing the human genome empowers translational medicine, facilitating transcriptome‐wide molecular diagnosis, pathway biology, and drug repositioning. Initially, microarrays are used to study the bulk transcriptome; but now short‐read RNA sequencing (RNA‐seq) predominates. Positioned as a superior technology, that makes the discovery of novel transcripts routine, most RNA‐seq analyses are in fact modeled on the known transcriptome. Limitations of the RNA‐seq methodology have emerged, while the design of, and the analysis strategies applied to, arrays have matured. An equitable comparison between these technologies is provided, highlighting advantages that modern arrays hold over RNA‐seq. Array protocols more accurately quantify constitutively expressed protein coding genes across tissue replicates, and are more reliable for studying lower expressed genes. Arrays reveal long noncoding RNAs (lncRNA) are neither sparsely nor lower expressed than protein coding genes. Heterogeneous coverage of constitutively expressed genes observed with RNA‐seq, undermines the validity and reproducibility of pathway analyses. The factors driving these observations, many of which are relevant to long‐read or single‐cell sequencing are discussed. As proposed herein, a reappreciation of bulk transcriptomic methods is required, including wider use of the modern high‐density array data—to urgently revise existing anatomical RNA reference atlases and assist with more accurate study of lncRNAs.

## Graphical abstract

Transcriptome‐wide molecular profiling of cells or tissue is used for multiple purposes including classification, pathway biology and drug repositioning. Replacement of microarray technology by short‐read RNA sequencing is premised on its superior performance. An equitable comparison reveals that the modern array yields a more reliable representation of the constitutively expressed transcriptome, while RNA‐seq does not accurately represent the long noncoding transcriptome nor pathway‐level modulation.

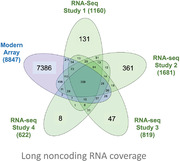

## 1 Introduction

High‐throughput profiling technologies are increasingly used to define the molecular repertoire of disease at the (epi)genomic, transcriptomic, proteomic, and metabolomic level.^[^ ^1^, ^2^, ^3^, ^4^, ^5^, ^6^, ^7^, ^8^, ^9^, ^10^, ^11^, ^12^, ^13^, ^14^ ^]^ Such technologies can be used to establish that patients, normally categorized to a single disease, display molecular heterogeneity;^[^ ^15^ ^]^ this may be linked to prognosis or be used to select more active drug combinations.^[^ ^16^, ^17^, ^18^, ^19^, ^20^ ^]^ Transcriptomics has led to various tools for stratified medicine^[^ ^14^, ^21^, ^22^, ^23^, ^24^ ^]^ by identifying signatures that better match patients to drugs.^[^ ^2^, ^25^, ^26^, ^27^, ^28^, ^29^, ^30^ ^]^ Typical statistical analyses applied to clinical transcriptomics data includes differential gene expression (DE) based on group mean differences;^[^ ^8^, ^31^, ^32^ ^]^ regression analyses of transcript abundance versus clinical status;^[^ ^2^, ^6^, ^30^, ^33^, ^34^, ^35^, ^36^ ^]^ and classification approaches.^[^ ^9^, ^37^, ^38^, ^39^, ^40^, ^41^, ^42^, ^43^, ^44^ ^]^ Regression and DE analyses are then used to identify the molecular pathways regulated between groups.

Capturing RNA expression is not done merely as a surrogate for measuring protein abundance;^[^ ^45^ ^]^ although this can be done when applied appropriately.^[^ ^46^, ^47^, ^48^ ^]^ Rather, RNA expression acts as a “biosensor”, integrating environmental,^[^ ^8^, ^31^, ^36^, ^49^ ^]^ epigenetic, and genetic^[^ ^50^, ^51^, ^52^ ^]^ influences. Modeling transcriptomic data provides information about the activity of proteins in a pathway, for instance, transcription factor status.^[^ ^53^, ^54^ ^]^ While protein abundance may not inform on protein activity,^[^ ^55^, ^56^ ^]^ transcriptome analysis can identify protein activity across networks and canonical pathways^[^ ^35^, ^36^, ^54^ ^]^ and thus extends beyond a simple surrogate for protein abundance. Each multi‐omic technology captures distinct and shared information, while each also introduces its own specific sources of variance. The recent trend to report that global transcriptomics fails to capture biology identified only by proteomics^[^ ^57^ ^]^ can be explained by poorer quality transcriptomics,^[^ ^35^ ^]^ including use of relative count RNA‐seq data, a lack of consideration of temporal relationships between transcription, translation, and proteostasis or the reliability of pathway or network models used to report differences.^[^ ^58^ ^]^ In general, studying any mismatch between RNA and protein requires a more equitable and balanced view on the limitations of all the laboratory methods employed.^[^ ^59^ ^]^ Thus, while the trend towards integrating multi‐omic data continues,^[^ ^60^, ^61^, ^62^, ^63^ ^]^ in a translational medicine setting, a single robust technology may deliver sufficient information and use fewer resources when applied to disease diagnosis or prognosis.^[^ ^64^ ^]^ For these reasons, transcriptome profiling will remain at the forefront of precision and stratified medicine efforts.^[^ ^14^, ^24^, ^65^, ^66^, ^67^, ^68^ ^]^

Herein, we reflect on the methods used for transcriptomics applied to translational medicine studies, including the reliability to cover the expressed transcriptome and the validity of pathway analysis. Processive technologies must ensure good reproducibility with comprehensive and accurate quantification of the transcriptome to ensure down‐stream analysis is reliable. Large‐scale tissue/blood sample acquisition from clinical trials or from biobank initiatives are costly, especially if accompanied by deep clinical phenotyping. We focus on the merits of the two main bulk transcriptome technologies (RNA‐seq and modern arrays) and consider these in light of whether there is limited clinical materials and study costs. In doing so, we seek to rebalance what we consider to be the overly “enthusiastic” introduction of short‐read RNA‐seq,^[^ ^69^ ^]^ and the persistent overly pessimistic view of arrays.^[^ ^70^ ^]^ We do not consider 3rd generation (long read) RNA sequencing methods in detail^[^ ^71^, ^72^ ^]^ as they are costly with limited throughput, representing basic research tools with very fluid and evolving laboratory and informatics protocols. We do not discuss single‐cell sequencing technologies^[^ ^73^, ^74^, ^75^, ^76^ ^]^ for similar reasons. Notably, the myriad of distinct single‐cell methods provide fractional (and often 3’ directed) coverage of the transcriptome, dominated by higher abundance genes,^[^ ^77^ ^]^ and thus provides a biased view of the single‐cell transcriptome.^[^ ^78^ ^]^ Such limitations affect the validity of any pathway analyses^[^ ^79^, ^80^, ^81^, ^82^ ^]^ representing an issue shared with 2nd generation short‐read RNA‐seq assays.^[^ ^83^ ^]^ The reader is directed elsewhere to in‐depth discussions of the biases inherent to single‐cell methods.^[^ ^84^ ^]^

## 2 Historical Overview of Gene Expression Profiling Methods from Bulk RNA Samples

Technological advances in the latter half of the 20th century made characterization of the tissue transcriptome possible, most notably the ability to “sequence” DNA to define the human genome. Nucleic acid sequencing began in the 1960s with decrypting the 76 nucleotides of alanine tRNA, a project that took over three years.^[^ ^85^ ^]^ Over a decade later, Sanger introduced “chain‐terminating” sequencing, enabling similar results in days.^[^ ^86^ ^]^ First‐generation sequencers employed a modified version of the Sanger method, permitted parallel running of 96 reactions and ≈200 bases per sample, to be sequenced per hour.^[^ ^87^, ^88^, ^89^ ^]^ This approach is highly accurate and can remain a method of choice for diagnosing rare diseases. Today, Affymetrix array and short‐read RNA‐seq are the two most widely used approaches for gene expression profiling of clinical samples (**Table** **1**). These methods both relied on the genome sequencing revolution—and profile RNA extracted from a few milligrams of tissue, ideally using between ≈500 ng (Affymetrix) to a microgram (RNA‐seq) of total RNA. In comparison, direct long‐read RNA sequencing, using Nanopore technology, still requires up to ten times more RNA.^[^ ^90^ ^]^

**Table 1:** Comparison of the general properties of modern high‐density arrays and short‐read RNA‐seq

Platform	High‐density array (e.g., Clariom D or HTA 2.0)	Short‐read sequencing (e.g., Illumina NovaSeq 6000)
Laboratory details		
Head‐line costs for like‐for‐like analysis^a)^	$300	>$750 >150 M ($300 for 30–50 M is typical option)
Typical “Read depth”^a)^	Estimated = 150 M paired‐end reads	20M‐60 M paired‐end (or >100 M paired for de novo assembly)
Typical recommended amount of RNA	>100 ng (strand specific kit)	>500ng
High abundance RNA depletion	Not required	YES (Ribosomal and/or Globin)
Samples bar‐coded to multiplex^b)^	N/A	YES (up to 24 per flow cell lane); however, multiplexing has caveats
RNA to DNA protocol	Linear amplification, including amplification‐free options, cRNA	Most commonly, 18‐cycle PCR non‐linear amplification to produce cDNA
Lab protocol	24 samples per workstation for high‐density array	24 bar‐coded samples per flow cell lane
Throughput per workstation	Medium‐high (lower density arrays can be run 384‐well format)	Medium (>60–150 M paired using S4 flow cell at 100–150 bp per read)
Typical run time per workstation (24 samples)	≈24 h	>48‐72+ h
Data file produced	Original image, CEL file	No original base‐image‐file; FASTQ
Method	Fragmented labeled cRNA hybridized to 7 million, multi‐copy, 25‐mer “probes”	Ligated fragmented cDNA hybridized to flow‐cell, in situ amplification and probabilistic base calling using digital imaging
Published technical performance		
Base call accuracy	N/A	>80% called near‐perfect accuracy (Q30)
Annotation accuracy	Each 25‐mer probe aligned or rejected to current genome/transcriptome	Sequence aligned to current genome/transcriptome using a predictive model
Relies on reference sequence	Yes (CDF); updated partial re‐alignment using updated CDF	Mostly; re‐alignment possible (> time/costs)
Reproducibility for the most abundant transcripts	R2 > 0.9	R2 > 0.9
Signal characteristics of raw data	Continuous signal, normally distributed (log), background low compared with signal	Discrete count data, with many missing values
GC correction of signal	YES	YES, possibly^[^ ^110^ ^]^
Sensitivity and dynamic range^c)^	Good dynamic range (up to 8000% for max vs min value for a probe‐set across clinical samples)	Highly sensitive as long as the cDNA for a transcript is represented in the library
Zero counts (missing data across biological replicates)	None (at the probe‐set level)	Frequent for >50% of the genome
Coverage of Protein‐coding RNA in sample	>90%	>80%
Coverage of non‐coding RNA in sample	>75%	Highly variable, <25%
Allele specific quantification	Not possible	Possible, with limited examples so far
Informatics related		
Raw data storage (CEL/BAM)^d)^	Up to 80 MB/sample	>1GB/sample (compressed)
Complete basic data analysis (from CEL or FASTQ file to pathway analysis)	≈3–7 days	≈weeks to months depending on how many analytical options are combined to explore the validity of the primary analysis
Normalization and quantification	Stream‐lined, validated with few variations^[^ ^30^, ^84^, ^99^ ^]^	Thousands of potential combinations; no gold‐standard^[^ ^115^, ^153^, ^251^, ^252^ ^]^
Differential expression	Established, validated methods^[^ ^129^, ^130^, ^131^ ^]^	Methods remain work in progress, with popular methods appearing problematic larger sample sizes^[^ ^153^ ^]^

^a)^ It is estimated that for RNA sequencing to quantify DE of lower expressed genes to the same degree as modern arrays, 150 million^[^ ^98^ ^]^ or up to 1 billion reads may be required^[^ ^151^ ^]^

^b)^ Multiplexing samples in a pooled sequencing library (with bar‐codes) assumes that the concentration of each library (cDNA) is relatively even. Clearly, if one sample or more has less rRNA depletion or is added disproportionately, this changes this condition. Further, if free barcoded adapter/index primers are present in a multiplexed pool, the free adapter has the potential to prime and extend library molecules in the same lane during the clustering step, which would result in misassignment of reads through index swapping. This can cause errors in demultiplexing data, as reads from one sample have the potential to end up in the FASTQ files of a different sample (information taken from https://www.med.stanford.edu/gssc/hiseq4000issue.html)

^c)^ There is no doubt that sequencing can be very sensitive, yet prior discussions of the dynamic range have been misleading. If a gene did not make it into most libraries or the depth of sequencing was insufficient to accurately quantify the gene in the baseline sample estimates of the increased gene expression in the post‐intervention samples suffer from being divided by a very small number. RNA sequencing is also subject to producing “zero” count values in many samples, diluting the mean group value for the denominator, which can dramatically inflate fold change values. The exact threshold for detection will be unclear and will vary depending on the choice of library and sequencing protocols

^d)^ Studies to compress raw data to make storing RNA‐seq raw data more affordable have identified significant challenges or artefacts^[^ ^253^ ^]^ and ultimately do not represent storage of original raw data—in contrast to the files produced during scanning of an array.

### 2.1 Introduction to Microarrays

Various array designs emerged in the 1990's, each with various designs and technical capabilities^[^ ^91^, ^92^ ^]^—with the Affymetrix array emerging as the most popular.^[^ ^92^, ^93^ ^]^ Arrays contain a “lawn” of oligonucleotide probes immobilized on a solid glass surface that bind complementary DNA molecules, that is, it is a dedicated, non‐competitive quantification strategy (i.e., each nucleic acid sequence has its own “detection” system). A biotinylated “sandwich” assay then yields a fluorescent signal in proportion to the concentration of the copy DNA (cDNA)—a facsimile of RNA as a strand‐specific DNA.^[^ ^93^ ^]^ Early Affymetrix array designs required 5 “chips” per clinical sample to cover the complete draft of the transcriptome.^[^ ^8^ ^]^ Each probe was designed to measure the 3’ end of transcripts. The drive to unravel the complexity of transcriptome, combined with the availability of the draft genome sequence, led to the development of the “chromosome tiling” array^[^ ^94^ ^]^ which studied transcription from the entire chromosome or genome.^[^ ^95^, ^96^, ^97^ ^]^ However, these were costly and never intended as a routine tool to quantify transcript abundance.

Modern high‐density arrays, relying on ≈7 million probes to cover the known transcriptome, became available in 2015^[^ ^98^ ^]^ and provide equitable profiling of the coding and non‐coding transcriptomes.^[^ ^2^, ^30^, ^54^ ^]^ Informatic analysis of this technology involves bringing together the signal from multiple copies of 25‐mer probes, distributed across the array surface. The probe level signal is assembled by combining a minimum of three probes into “probe‐sets”. The exact composition of each probe‐set is defined by reference to a “map” called a chip definition file (CDF); which is routinely configured to exon or transcript level resolution.^[^ ^54^ ^]^ Regardless of when the array was designed, the design of the CDF can be updated regularly to ensure this process remains accurate.^[^ ^99^ ^]^ The physical design of the array can also be updated as the transcriptome becomes more complete. The raw data from each array experiment is retained as a binary “CEL file”. For very large‐scale projects, the high throughput lower density “peg” array may be useful, processing clinical samples in 96‐ and 384‐well format using a GeneTitan workstation (Thermofisher Inc). This format provides coverage of the known protein‐coding transcriptome, but with limited resolution, while the equivalent non‐coding array remains to be developed.

Modern high density arrays generate a signal ranging from <1 to 16 on the log2 scale.^[^ ^2^, ^30^, ^100^ ^]^ Through cross‐reference with the literature, the minimal signal for genuinely expressed genes appears to vary between 2 and 3 log_2_ units. By this criteria, low expressed genes essential physiological roles can be detected, for example, for muscle we observe expression of nicotinic acetylcholine receptor RNA from the neuromuscular junction.^[^ ^101^, ^102^, ^103^ ^]^ Identification of physiologically active, but low‐expressed genes, for each tissue type, along with statistical thresholds, is required to define the lower end of the biological signal. Further, a study‐specific probe‐level scan can be used to remove probes with aberrant signals, before probes are combined into transcript level CDF design.^[^ ^2^ ^]^ This study‐specific approach typically removes ≈20% of probes, while realignment—to check probe specificity—removes a further 15%. Thereafter, greater than 90% of the remaining probe‐sets produce a signal above the empirical threshold of between 2 and 3 log_2_ units.^[^ ^2^, ^30^, ^54^ ^]^ The signal from each probe on the high‐density arrays can also be scaled by their guanine and cytosine (GC) content (Thermofisher Inc, SST‐GCCN Whitepaper) by processing the CEL file. This process makes the signal generated more comparable across genes (further distinguishing this data type from RNA‐seq based relative counts). In the end, about 5 from 7 million probes are typically used to detect ≈500,000 exons or ≈100 000 transcript probe‐sets in each human tissue type, using a Human Transcriptome Array (HTA) 2.0 array.^[^ ^54^ ^]^ Transcript signals can be further summarized in a targeted manner for statistical analysis; for example, the signal originating only from the untranslated region can be extracted and compared across conditions, identifying regulatory events unseen with conventional gene level analysis.^[^ ^35^ ^]^ Finally, it should be noted that the quantification of very short transcripts (<30 base pairs (bp)) by an array will not be ideal as such a signal would rely on less than three distinct probes.

### 2.2 Introduction to Short‐Read RNA‐Seq

Next‐generation sequencing short‐read RNA‐seq emerged in 2008, reflecting advances in several laboratories.^[^ ^104^, ^105^ ^]^ An approach developed by Solexa (acquired by Illumina) generates most of the sequencing data deposited on the Sequence Read Archive (SRA) and thus is the focus of this review.^[^ ^106^ ^]^ Short‐read RNA‐seq requires making a DNA copy of RNA before analysis, after which in situ cluster formation by amplification is tracked. DNA clusters are generated on a flow cell using a modification of PCR, called bridge amplification, where DNA polymerase directs chain elongation from DNA templates using reversible chain‐terminating nucleotides. Each cluster of amplified nucleotides is then “read” – typically via a four‐color system for base calls—to determine sequence information.^[^ ^106^, ^107^ ^]^ Short‐read RNA‐seq has several laboratory requirements that array protocols do not, such as initial depletion of the most abundantly expressed genes (e.g., ribosomal RNA—rRNA) which if not removed, dominates most of the sequence reads.

Short‐read RNA‐seq “counts” are based on counting reads of 50–200 bases, and gene abundance is inferred from the number of counts that map back to a given gene,^[^ ^108^, ^109^ ^]^ scaled to the total number of mapped reads in the sequencing run. Mapping each signal to an individual gene is mostly done using a reference transcriptome/genome. Allocation of multi‐mapping reads is achieved using probabilistic‐based estimates, which remains a source of bias and uncertainty.^[^ ^70^ ^]^ Counts represent the relative expression of the gene within the cDNA library during a single sequencing run.^[^ ^76^ ^]^ Longer genes yield more reads, but other factors such as nucleotide content also influence the number of reads counted,^[^ ^110^ ^]^ rendering the quantification of transcripts a nontrivial challenge.^[^ ^111^ ^]^ The influence of gene length on data processing can artificially result in significant pathways in down‐stream statistical analysis.^[^ ^112^ ^]^

Laboratories specializing in genomics, carry out complex “de novo” assembly of sequencing data to discover novel transcripts, often in non‐mammalian species without completed reference genomes.^[^ ^113^ ^]^ Here, reads are assembled using various complex models, with or without the use of a genome from a related species. While any initial alignment can be quick,^[^ ^114^ ^]^ the complete process is iterative^[^ ^108^ ^]^ and may take weeks or months fully evaluate the validity of the modeling choices. Such analyses are not part of the standard transcriptomics service, while the lack of precise reporting of the hundreds of potential experimental options^[^ ^115^ ^]^ for RNA‐seq limits meaningful replication.^[^ ^116^ ^]^ Thus, despite the potential capacity for transcript discovery,^[^ ^69^ ^]^ most short‐read RNA‐seq studies are not designed to report novel transcripts.^[^ ^30^, ^70^ ^]^

## 3 Performance Metrics for Short‐Read RNA‐seq versus Modern High‐Density Arrays

Several influential articles compared RNA‐seq with (older) array technology,^[^ ^69^, ^105^ ^]^ explicitly reflecting on the “death of the microarray”^[^ ^117^ ^]^ and these have fueled a persistent inaccurate view of arrays.^[^ ^70^ ^]^ To re‐examine this topic, it is critical to contrast the most common short‐read RNA‐seq method with the latest modern high‐density array and reflect on empirical performance rather than any hypothetical advantages. Using a variety of published datasets produced from human clinical samples (in this case snap‐frozen human skeletal muscle) we present easy‐to‐follow head‐to‐head comparisons. We have reported comparable observations using post‐mortem human brain as well as from snap‐frozen RNA form blood and adipose tissue, elsewhere.^[^ ^2^, ^28^, ^30^, ^54^, ^57^, ^118^, ^119^, ^120^ ^]^

For a gene profiling technology to be useful for modeling disease biology (pathways or networks), it must capture the complexity of the transcriptome with limited bias, and that bias must be quantifiable. To have cost‐effective clinical utility, it should be reproducible, generate consistent data comparable to a reference, and have a quick turnaround cycle involving robust computational pipelines. Costs of raw data storage should also not be overlooked. Short‐read RNA‐seq produces gigabytes of summarized data per sample, while each modern array raw data file is 70 MB. While short‐read RNA‐seq technology benefits from efforts to make all forms of DNA sequencing more cost‐effective^[^ ^121^ ^]^ these improvements have not led short‐read RNA‐seq to become cost effective compared with the modern array, reflecting longer laboratory and informatics processing times than arrays (Table 1).

A highly influential 2009 review (with > 13 500 citations) provides the basis for the often‐stated superior performance of RNA‐seq.^[^ ^69^ ^]^ It highlighted a comparison with the now discontinued BeadChip array (probes for ≈13 000 genes) and, unsurprisingly, found that RNA‐seq detected ≈25% more genes.^[^ ^105^ ^]^ Yet had an old Affymetrix U133+2 array been used; it would have performed as well as RNA‐seq at this time. A detailed comparison^[^ ^69^ ^]^ was made with the “chromosome tiling” array,^[^ ^94^ ^]^ reporting that RNA‐seq had less background noise, improved ability to differentiate between transcript isoforms, and greater dynamic range as well as better reproducibility for DE analysis.^[^ ^69^ ^]^ These comparisons are not terribly meaningful as the “chromosome tiling” array was not designed^[^ ^94^ ^]^ as a quantitative tool, nor does this comparison accurately reflect the performance of a modern array.

An in‐depth comparison of short‐read RNA seq and arrays was made by the Array/Sequencing Quality Control Consortium in 2014 (SEQC/MAQC‐III Consortium or SEQC for short) in a series of publications. SEQC considered sensitivity, transcriptome coverage and DE.^[^ ^122^ ^]^ SEQC utilized the Affymetrix U133+2 array (older array with 3’ biased probes) and compared performance with at the time state of the art RNA‐seq (which was not like‐for‐like in terms of cost). RNA‐seq unsurprisingly discovered novel exon‐exon junctions and transcripts versus what was possible with the older array. SEQC also reported that relative expression results were consistent among different RNA‐seq platforms, but only if specific ad hoc filters were used.^[^ ^122^, ^123^ ^]^ Notably, SEQC reported a comparable performance between RNA‐seq and the U133+2 array technology, with the best DE results produced by the U133+2 array (See Figure 3e in ref. [122]). These observations had little impact on how array performance was presented. Note that the data provided by the 2014 SEQC consortium study is summarized to “ref_seq” RNA identifiers and thus the total number of genes detected by RNA‐seq was at least 50% lower than claimed (See https://doi.org/10.5281/zenodo.7430956 for more details). In general, we believe that the conclusions reached in 2014 need to be revisited to accommodate comparison between typical RNA‐seq and modern high‐density array using best practices.^[^ ^2^, ^54^ ^]^

The SEQC publications did not discuss technical reproducibility of short‐read RNA‐seq in much detail nor did they study biological replicates.^[^ ^122^ ^]^ They did note that including genes with very high counts (top 10%) exaggerates any reproducibility calculation (See Figure S22, Supporting Information^[^ ^122^ ^]^). For the remaining transcriptome, technical reproducibility was reported as being *R* ^2^ = 0.7, similar for biological replicates using the HTA 2.0 array (*R* ^2^ = 0.8,^[^ ^2^ ^]^). Unfortunately, many studies still quote erroneous values for reproducibility. As mentioned above, specialized sequencing protocols will probably profile short RNAs (<50 nucleotides) better than arrays.^[^ ^125^, ^126^ ^]^ However, independent technical replicates are difficult to locate for this type of RNA‐seq data, and estimating consistency has numerous caveats. In general, discussion of technical reproducibility is rare in the literature. In addition, raw RNA‐seq count data can often be modified—prior to statistical modeling—adding a small positive signal to all values prior to log transformation. When applied to the extremes of signal abundance this can lead to misleading conclusions (See Figure 5 in Ma et al.^[^ ^124^ ^]^).

Does RNA‐seq deliver the promised unbiased coverage^[^ ^69^, ^70^ ^]^ of the transcriptome? According to the latest release from the Genome Reference Consortium, the human genome (GRCh38.p13) consists of >20 000 protein‐coding genes, >24 500 non‐coding genes,^[^ ^127^ ^]^ and >15 000 pseudogenes^[^ ^128^ ^]^ and these collectively give rise to >245000 possible transcripts. While >50% will show low or no level of expression in any given cell type, low‐expressed genes nonetheless serve vital roles in physiology, for example, transcription factors, signaling kinases and receptors. For RNA‐seq, a gene known to be expressed but not detected (false negative) in a cDNA library, returns a “zero value”; while a value will be generated for each probed expressed gene, in each sample, using the modern array, down to the level of detection of that platform. Existence of “zero values” across sample rows is an underappreciated problem for RNA‐seq,^[^ ^120^ ^]^ representing up to 50% of the data file for projects using typical sequencing depths (30–100 million read alignments). How those “zero values” are modelled is critical and can impact on estimates of variance within the dataset and thus influence false discovery rate (FDR) calculations.^[^ ^129^, ^130^, ^131^ ^]^ Missing values are problematic for classification and regression models because they modify the sample size being analyzed per gene. Further, if the aim is to test the performance of a multi‐gene classifier, the assessment in any individual sample may be compromised by the stochastic coverage achieved with RNA‐seq. If instead of raw count values, the investigator is provided “transformed” data by a core facility service, the complexity introduced by “zero counts” would be obscured.

**Figure 1:**
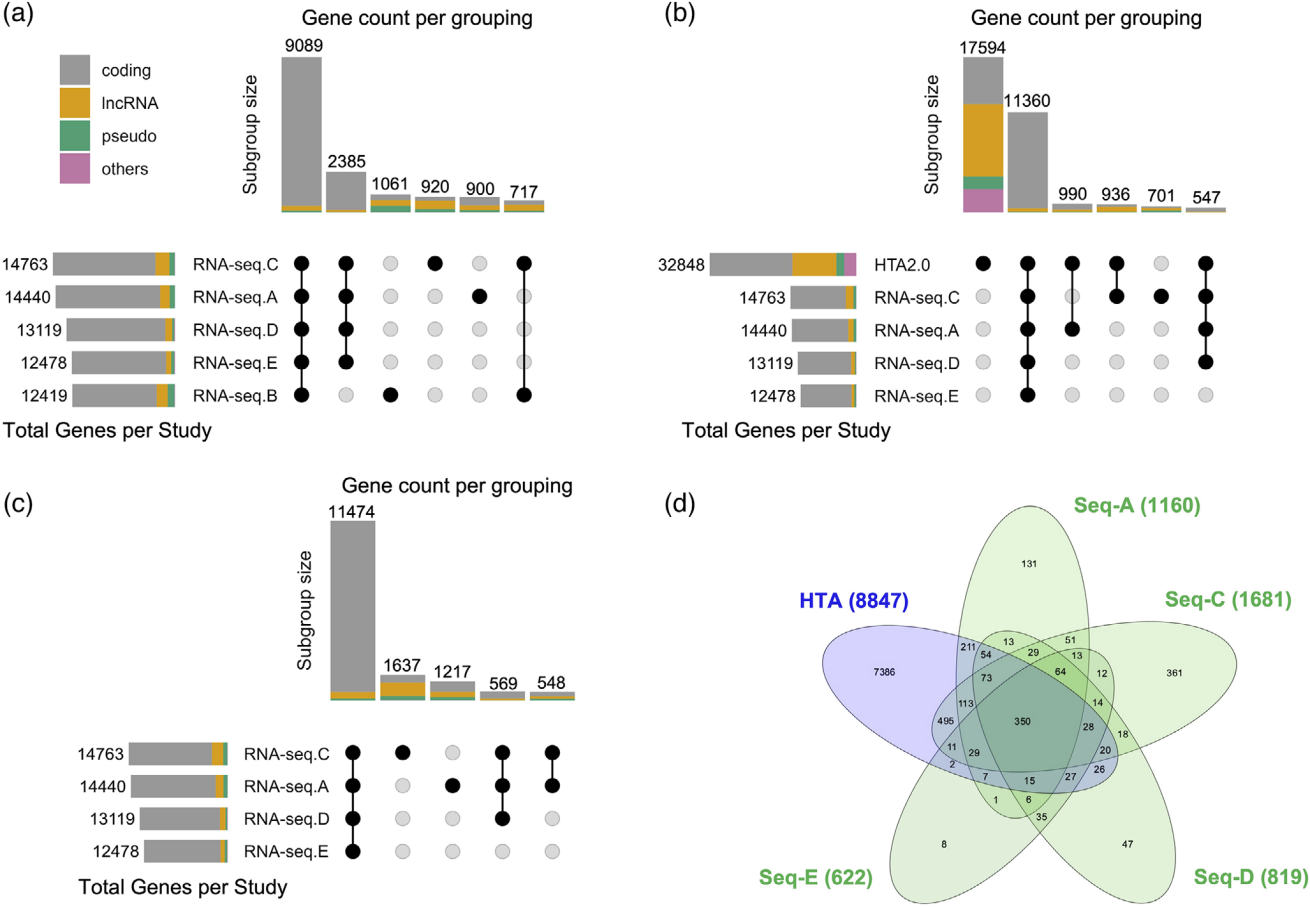
Coverage of the human muscle transcriptome. We consider the core (or constitutive) transcriptome for skeletal muscle from 810 sedentary humans. Five (*n* = 53‐278) short‐read RNA‐seq data sets profiling skeletal muscle (total = 619. RNA‐seq.A = FUSION cohort (≈60 million reads, *n* = 278,^[^ ^133^ ^]^ RNA‐seq.B = GESTALT cohort (≈60 million reads, *n* = 53,^[^ ^120^ ^]^), RNA‐seq.C = Robinson cohort (≈44 million reads, *n* = 74,^[^ ^57^ ^]^), RNA‐seq.D = Kulkarni cohort (≈34 million reads, *n* = 136,^[^ ^135^ ^]^) and RNA‐seq.E = Rubenstein cohort (≈29 million reads, *n* = 78,^[^ ^132^ ^]^)). A threshold for a gene being detected in a cDNA library typically relies on a threshold of a minimum of 5, 8, or 16 raw counts per gene^[^ ^122^ ^]^ and we have utilized >5 in the present analysis. To count as being constitutively expressed (e.g., tissue type defining) each gene should reach this threshold in every sample within a study. Modeling of disease genes can reduce this threshold to being detected in all case samples (for example). Defining the constitutive transcriptome is very critical for the interpretation of pathway analysis and so we focus on genes that were above background noise in each sample, within a given laboratory experiment to subsequently illustrate the impact on pathway statistics. The RNA‐seq data are contrasted with data produced using a modern high‐density array (HTA 2.0, *n* = 191^[^ ^2^ ^]^). A) Examination of the genes consistently detected per study using RNA‐seq and their overlap across studies, including major biotypes. Each cohort (A–E) has a further 10–15% of protein‐coding genes are expressed in every sample but unique to that RNA‐seq analysis B) As the RNA‐seq data set produced less reliable data, the core genes expressed using RNA‐seq (>95% of which are protein‐coding) are re‐plotted omitting RNA‐seq.B cohort. C) Comparison of the four RNA‐seq cohorts A,C–E) with the muscle HTA 2.0 array data. The array data is processed to remove all low‐performing probes (≈2 million), and then after summarizing at the probe‐set level (ENST), only probe‐sets with signals above 2 log2 units are retained, equating to >1SD absolute signal value.^[^ ^2^, ^30^ ^]^ This plot shows that the array captures a far more comprehensive view of the noncoding transcriptome. D) Application of a more conservative signal filter to the HTA data reduces the number of reported genes (some of which are genuine signals). The list of long noncoding RNAs (*n* = 8847) is contrasted with the other RNA‐seq data sets from (B) using a Venn diagram tool.^[^ ^136^ ^]^ Around 20% of those lost with this more severe arbitrary filter were in fact expressed in all samples form one of the RNA‐seq datasets. Further details of the code and input data can be found here https://doi.org/10.5281/zenodo.7430956.

To illustrate the constitutively expressed transcriptome for human muscle tissue, we produced plots for five RNA‐seq studies of human muscle tissue.^[^ ^57^, ^119^, ^120^, ^132^, ^133^ ^]^ Details of the code used, and input data can be found here https://doi.org/10.5281/zenodo.7430956. We found that between 12 000 and 15 000 genes are consistently detected in all samples from each individual study. Notably, only 8700 protein‐coding genes were expressed in all samples across all studies (**Figure** **1**A) while the expected figure should be closer to 16 000 protein coding genes for muscle tissue.^[^ ^134^ ^]^ After removing Study B, somewhat greater consistency was observed across the remaining studies (Figure 1B). Muscle tissue obviously does not express all protein‐coding genes, yet it is implausible that healthy human muscle expresses such a wide repertoire of study‐specific protein‐coding genes (Figure 1A). Further, only a small number of non‐protein‐coding genes were detected across these RNA‐seq studies and in an inconsistent manner. Thus, each study consistently detected a few thousand genes that were not consistently expressed in the other four studies and therefore the detectable transcriptome (or background) for the same tissue varies greatly between each RNA‐seq study. This variation has major implications for the validity and reproducibility of any subsequent pathway analysis (^[^ ^79^, ^82^ ^]^, see below).

Due to improvements in design since the 2014 SEQC studies,^[^ ^122^, ^137^ ^]^ modern arrays now cover most of the annotated transcriptome, detecting ≈10% more protein‐coding genes than RNA‐seq, in the example we present (Figure 1C). For noncoding RNAs, the difference between RNA‐seq and modern arrays is far more pronounced. Long noncoding RNA (lncRNA) can be human‐specific^[^ ^127^, ^138^ ^]^ modulate key epigenetic events^[^ ^139^, ^140^, ^141^ ^]^ and, despite their name, are increasingly noted to also contain atypical open reading frames,^[^ ^142^ ^]^ that code for novel short peptides,^[^ ^143^ ^]^ or catalytic RNAs.^[^ ^141^ ^]^ When RNA‐seq was used to define the human tissue transcriptome in 2015,^[^ ^144^ ^]^ we noted a major under‐reporting of lncRNA expression^[^ ^2^, ^54^ ^]^ in three human tissues.^[^ ^2^, ^30^, ^54^ ^]^ In fact, RNA‐seq consistently detects only ≈1000 lncRNAs in human muscle per study, and with moderate overlap between studies (Figure 1A,D). Arrays contain probes for nearly 10 000 lncRNAs using an ensembl‐based CDF map (Figure 1C) and most of the lncRNAs detected across the four RNA‐seq data sets are detected by the array. When a conservative signal detection filter is applied to the array data, there are still >8000 lncRNA detected (Figure 1D) and of those removed, 20% were detected in at least one of the RNA‐seq studies. Thus, routine short‐read RNA‐seq profiling of human tissue appears to miss most of the lncRNA transcriptome.

**Figure 2:**
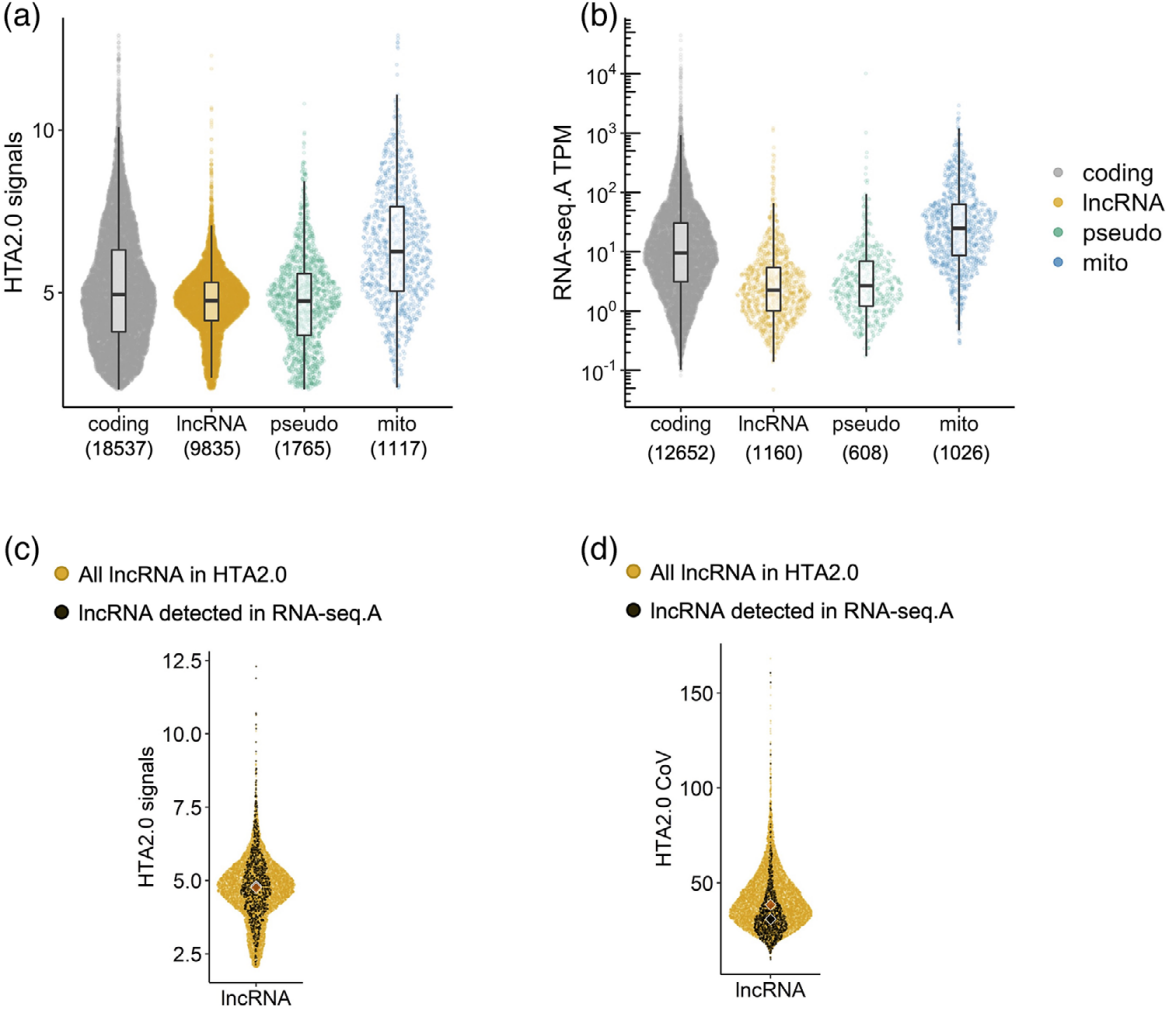
Distribution of abundance of different transcript classes. A) using the data from *n* = 191 human muscle samples^[^ ^2^ ^]^ profiled on the HTA 2.0 array, the distribution of the log2 signal is plotted (median, interquartile range, and full distribution) for protein‐coding (grey), lncRNA, pseudo‐, and mitochondrial genes. The average abundance of mitochondrial‐related genes is greater than other classes of RNA. B) Using the largest muscle RNA‐seq data set (RNA‐seq.A = FUSION cohort (*n* = 278,^[^ ^133^ ^]^) and the data processing steps described in Figure 1 legend, the same classes of genes are plotted—albeit the total number of genes is lower than for the HTA data. Scaling to transcripts per million (TPM)—normalized counts to gene length—indicates that RNA‐seq reports lncRNA expression as lower protein‐coding genes—comparison of count values across genes is not necessarily valid with RNA‐seq data. C) The lncRNAs detected by RNA‐seq are in fact a sample from across the range of abundances of lncRNA quantified by the array indicating abundance does not explain their detection by sequencing. D) The lncRNAs detected by RNA‐seq are a sample from across the range of cohort‐wide variation (CoV, coefficient of variation) of lncRNA quantified by the array. In C and D, the black “violin” represent the median value for the RNA‐seq detected, calculated from the array data, with the orange diamond reflecting the entire array lncRNA data. Further details of the code and input data can be found here https://doi.org/10.5281/zenodo.7430956.

Failure to be represented in the cDNA library is the most likely reason RNA‐seq data show poor agreement for lncRNAs across data sets and only a fraction of those detected by array (**Figure** **2**A–D). LncRNAs lack extensive poly‐A tails and are therefore not efficiently incorporated into cDNA libraries by routine RNA‐seq protocols.^[^ ^145^ ^]^ What drives the stochastic nature of lncRNA detection between comparable RNA‐seq studies? We illustrate that lncRNAs detected by RNA‐seq originate from across the range of expression values (array data) and thus are not just a subsample from higher abundant lncRNAs (Figure 2C). In fact, lncRNAs are routinely described as “low abundance transcripts”^[^ ^150^ ^]^ but this reflects a reliance on RNA‐seq data. Using the HTA 2.0 array and a probe‐filtered custom CDF^[^ ^2^, ^54^ ^]^ the abundance of lncRNAs is near‐identical to protein‐coding genes (Figure 2A). Other classes of non‐translated RNAs—such as pseudo genes—which act as miRNA decoys and regulatory molecules^[^ ^128^ ^]^—are also expressed with abundance not that distinct from protein coding genes (Figure 2A,B). A genuine low abundance gene, represented in a cDNA library, combined with very deep sequencing would result in counts, while an array might not report a signal (the modern array is able to detect transcripts form minor cell populations^[^ ^28^ ^]^—see below). This hypothetical benefit of RNA‐seq is not encountered in most clinical studies, as the cost of very deep sequencing is prohibitive and deep sequencing introduces other biases, compromising the analysis of the data (see below).

In contrast, RNA‐seq detected lncRNAs did tend to originate from those which exhibit less variable expression (array data) across muscle samples (Figure 2D). This more consistent expression may partly explain why they were detected consistently in all RNA‐seq profiles, for a given study—but not their lack of consistent expression across studies. A lack of appreciation of biases within RNA‐seq data has led to an increasing number of erroneous conclusions regarding tissue‐ and cell‐specific expression for lncRNAs.^[^ ^2^, ^146^, ^147^, ^148^ ^]^ This aspect of transcriptome coverage also impacts on pathway analysis of single‐cell sequencing, as most methods to date quantify three or four thousand transcripts per cell. Biased in high abundance RNA from metabolic genes, this has implications for the nature of sub‐clusters of cells based on gene expression.^[^ ^73^, ^149^ ^]^ In summary, cDNA libraries produced by RNA‐seq protocols appear to have stochastic properties, ensuring genes are missing seemingly at random, such that in practice modern arrays provide broader and less biased—by biotype—coverage of the transcriptome.

**Figure 3:**
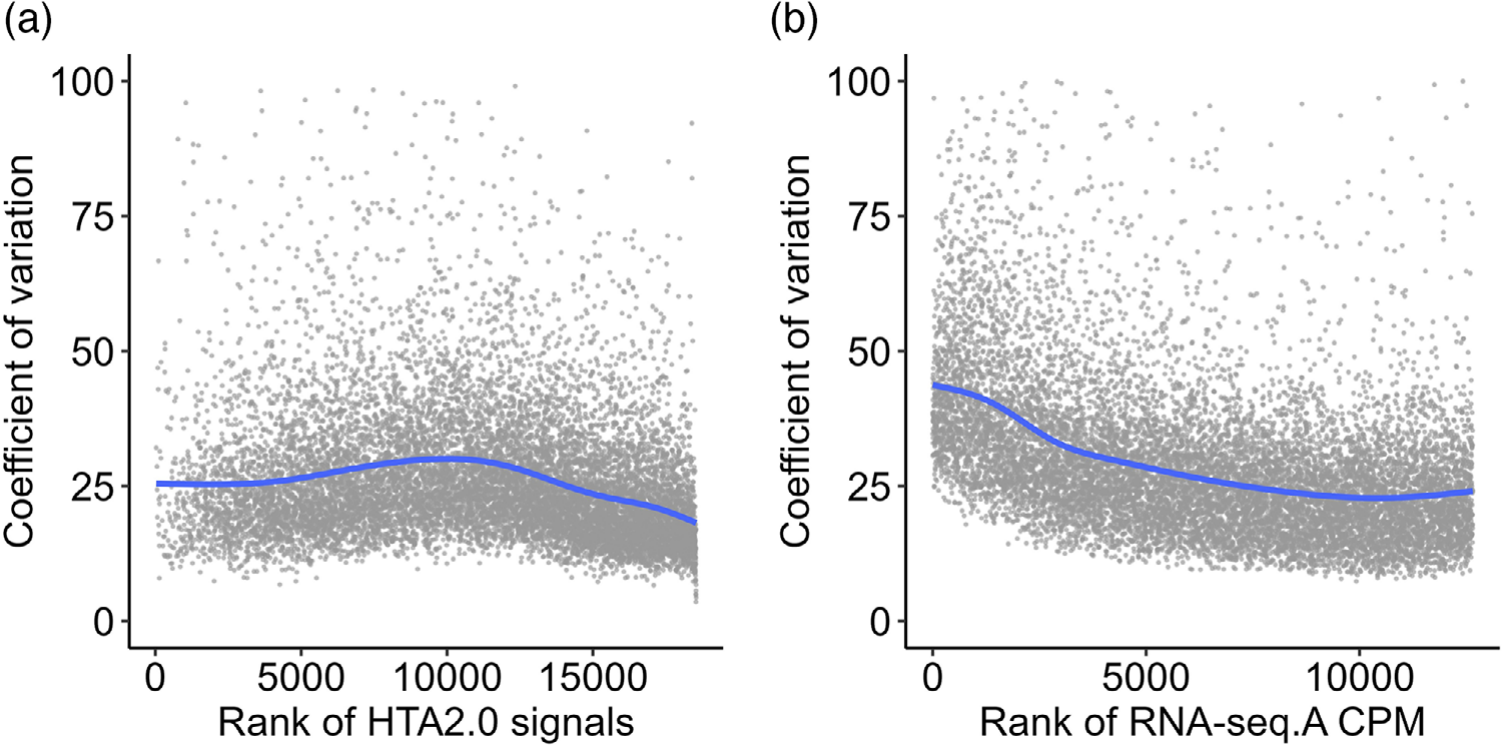
The relationship between protein‐coding gene abundance and the cohort‐wide coefficient of variation for gene expression. HTA 2.0 data used in Figures 1, 2, 3, and the RNA.seq data set A (FUSION cohort). A comparison of the coefficient of variation for each gene was made against the rank order abundance to determine if variation was largely related to the detectable abundance. A) Using array data, the coefficient of variation for each protein‐coding gene (common to both studies) versus the rank order of intensity values for gene expression is plotted. B) Using RNA.seq data, the coefficient of variation for each protein‐coding gene (common to both studies) versus the rank order of intensity values for gene expression is plotted. Note that 43 (HTA) and 89 (RNA‐seq) extreme CoV values are not in plot axes. Further details of the code and input data can be found here https://doi.org/10.5281/zenodo.7430956.

The ability of each method to reliably model DE is also important. Coverage is vital in this context; however, variance in gene expression (biological and technical) is key. Variance is not equally distributed across the rank order of counts (RNA‐seq) or signal (array), and thus power to detect DE modeling will be influenced by gene “abundance”. It is estimated that for RNA sequencing to quantify DE of lower expressed genes to the same degree as modern arrays, 150 million^[^ ^98^ ^]^ or even up to 1 billion reads may be required.^[^ ^151^ ^]^ For protein‐coding genes, commonly detected in both RNA‐seq and HTA 2.0 array, the coefficient of variation (CoV) for the RNA‐seq data is greater than observed using the array (**Figure** **3**), particularly as the rank of normalized count values decrease (Figure 3B). This relationship is not seen with array data (Figure 3B), implying there is less bias in quantifying DE using the array (at least in this example).

Data generated by modern arrays or short‐read RNA‐seq differs in other substantial ways. Observations presented above indicate that in practice the modern array detects more of the tissue transcriptome, with less bias (biotype and abundance). Greater variation, with RNA‐seq, should convey a disadvantage in detecting DE, yet the SEQC reported that RNA seq outperformed old arrays for drug‐induced DE,^[^ ^137^ ^]^ particularly for genes expressed at low levels. However, the opposite has since been reported when RNA‐seq was compared with a modern array, with the HTA 2.0 array detecting ≈25% more DE than RNA‐seq—leading to the discovery of more regulated pathways.^[^ ^145^ ^]^ The same authors noted that several low‐expressed protein‐coding genes detected by the array were not detected by RNA‐seq.^[^ ^145^ ^]^ Recent modelling concluded that modern arrays quantify “low signal” genes more accurately,^[^ ^98^ ^]^ with highly abundant genes being assayed more equivalently. Indeed, DE analysis of abundant genes will generally produce similar conclusions when comparing arrays with RNA‐seq^[^ ^118^ ^]^ yet this sort of outcome does not satisfy the intended purpose or utility of global transcriptomics. Other recent reports have also concluded that modern high‐density arrays are more sensitive;^[^ ^152^ ^]^ and this is before any added benefit of using an optimized CDF design. Thus, any claim that RNA‐seq is superior at detecting DE—in a like‐for‐like setting—is probably incorrect, especially for the many genuinely expressed genes, it fails to detect. Systematic differences between the occurrence of zero counts between cases and controls requires special attention in DE analysis of RNA‐seq data, given that zero counts are not synonymous with “not expressed”, and the profound influence zeros (or “adjusted” zeros) have on any estimation of group mean variance.

**Figure 4:**
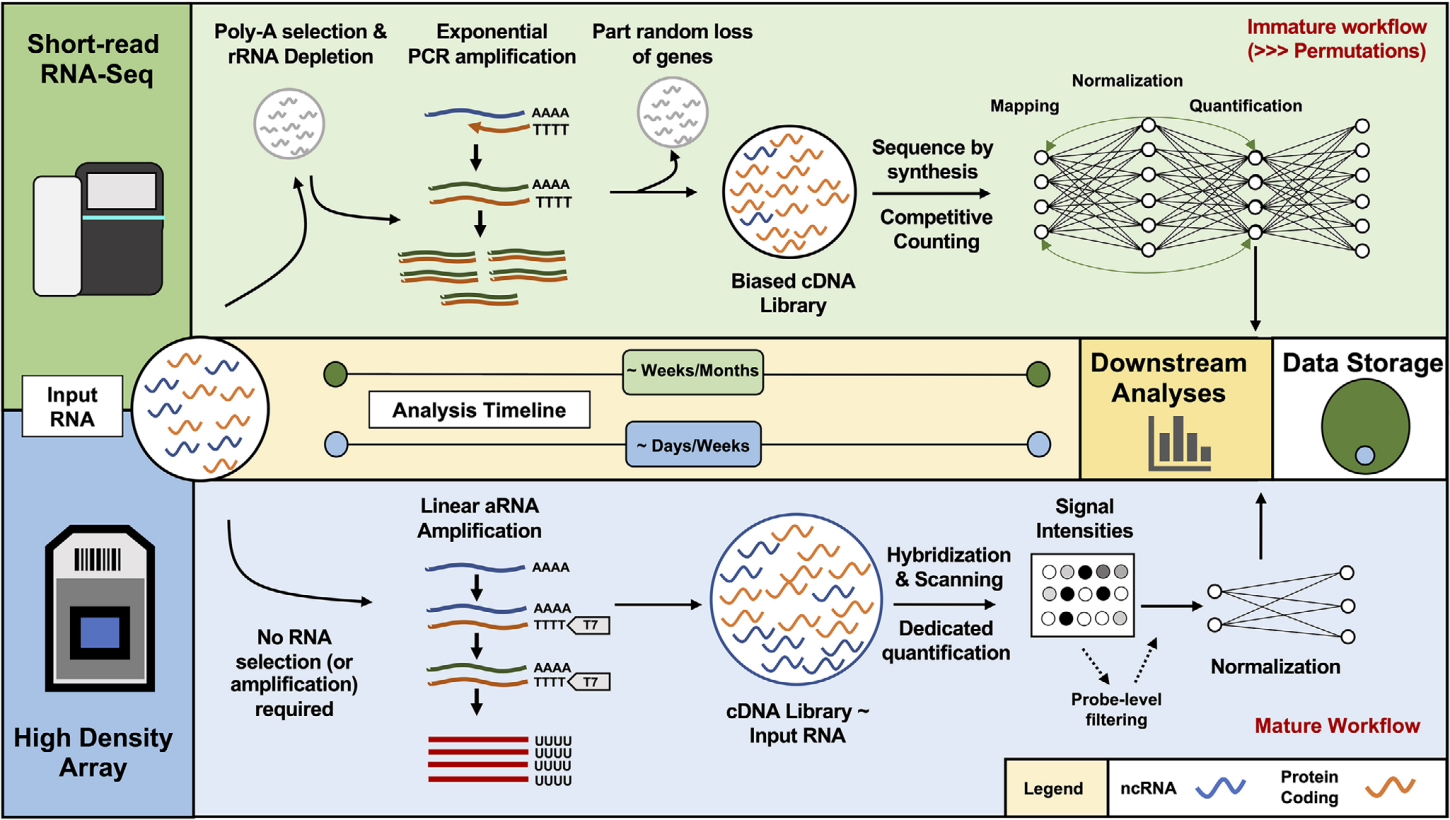
Summary of the transcriptomics workflow; from sample processing to statistical analysis, and storage of raw data.

Thus, so far, we have noted that many transcriptomic articles often begin with restating that RNA‐seq produces less biased data and are more sensitive than microarray‐based methods,^[^ ^70^ ^]^ claims that are partly attributable to early inequitable comparisons^[^ ^69^ ^]^ and claims that now should be retired. In practice, the modern high‐density array offers superior coverage for similar costs, and in the following sections, we cover some of the reasons that underpin these empirical observations (Figures 1, 2, 3). We highlight why coverage that is broad and of limited bias determines the validity of downstream pathway analysis—something very relevant for correctly modelling single‐cell sequencing data. We also note that the array relies on a more standardized informatic workflow, using methods found to be robust,^[^ ^152^, ^153^ ^]^ and produces raw data files which have much lower long term storage requirements (**Figure** **4**).

## 4 Key Differences between Transcriptome Profiling Technologies

### 4.1 Composition of the cDNA Library and Depth of Sequencing

Short‐read RNA‐seq profiles a cDNA library (not RNA) and this cDNA library is not a complete representation (“coverage”) of the transcriptome. Even exceptionally deep (and costly) sequencing will only detect a gene if a copy is present in the cDNA library.^[^ ^152^, ^154^, ^155^ ^]^ Coverage is sensitive to RNA degradation, and signals from inferior cDNA cannot be salvaged by deeper sequencing.^[^ ^156^ ^]^ If a low expressed transcript is “jackpotted” during cDNA synthesis – an early, random selective PCR amplification event—then deeper sequencing can further exaggerate this bias. Estimates have been made for the necessary sequencing depth per experiment, but these will be context specific (genes of interest, library quality etc.), and reassurances that 20–50 million read alignments are sufficient will not cover every situation. Characterizing array performance as an equivalent depth of sequencing is also difficult. For example, ≈65 million reads are reported to match the older 3’ Agilent array^[^ ^157^ ^]^ and 40 million aligned reads for the old U133+2 array.^[^ ^123^ ^]^ To match the HTA 2.0 Affymetrix array, the required read depth has been estimated to be >150 million paired‐end reads (ThermoFisher.com). The fact that the HTA 2.0 array provides quantification for >50% more transcripts than 50 million paired‐end reads (Figure 1C), illustrates that these estimates are not reliable. Variations in “effective” sequencing depth is mostly ignored, that is the depth of sequencing for most expressed genes, after consideration of the extreme number of counts attributed to the few very high abundance genes, for example, in muscle this would be mitochondrial genes.

If the background transcriptome (constitutively expressed protein coding genes) is not easily defined or varies between replicate studies (Figure 1) then ontology pathway analysis will have questionable validity and reduced comparability across studies.^[^ ^79^, ^82^ ^]^ Failure for a genuinely expressed gene to make it into an RNA‐seq cDNA library results in a “zero count” entry in the data file—providing the exact same profile as a gene that is not expressed. Visual inspection of raw count data reveals that “zero counts” predominate for ≈50% of the genome in any given RNA‐seq study. A small positive value can be added before the transformation of counts to other units, results in obfuscating these “zeros”. This type of standard data manipulation complicates the estimate of variance across the data^[^ ^158^, ^159^ ^]^ impacting for example, on permutation‐based FDR calculations.^[^ ^131^ ^]^ It is challenging to know if the “zero counts” represents a failure in the cDNA library, a genuine lack of expression or insufficient depth of sequencing. For lncRNA, we show that lack of detection almost certainly reflects a failure in the cDNA step, with limited consistency and low coverage across five independent RNA‐seq studies, yet most “RNA‐seq detected lncRNAs” are detected using the array (Figure 1D).

**Figure 5:**
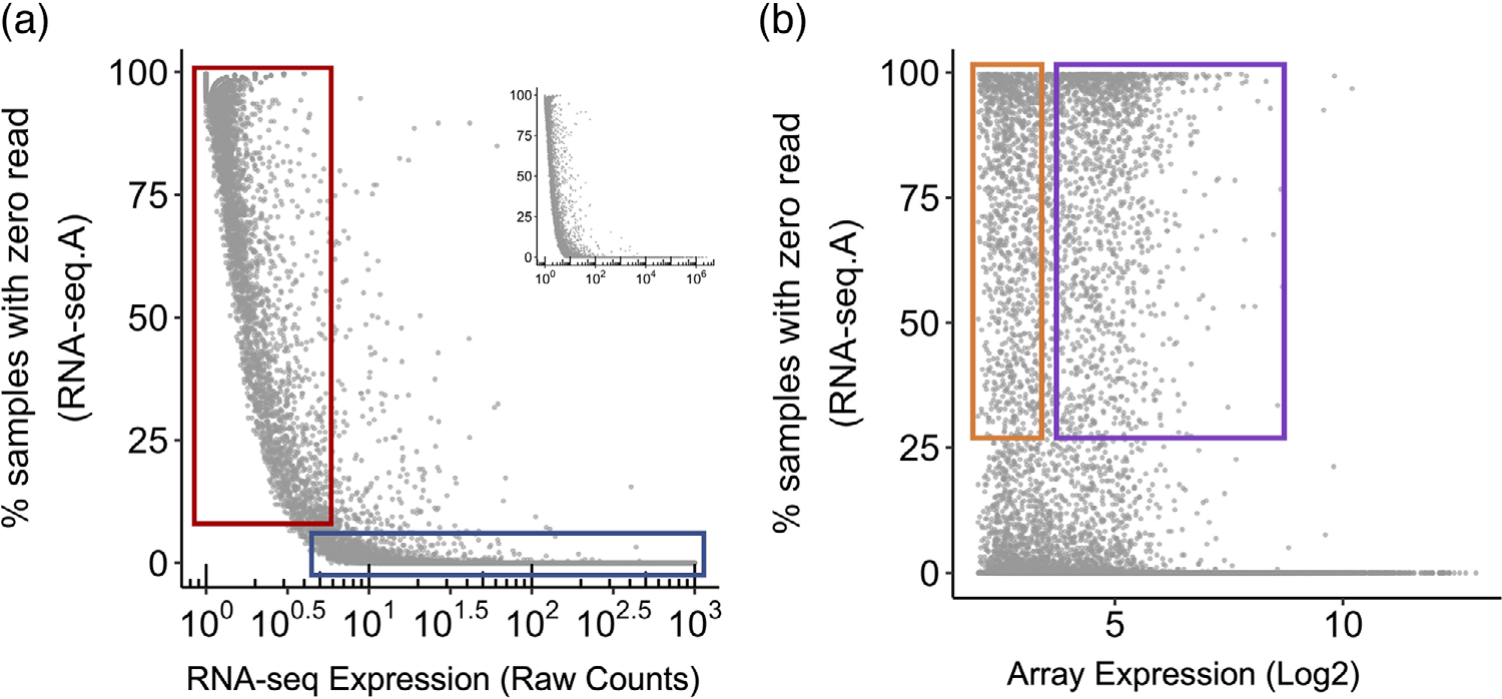
The relationship between protein‐coding gene levels and the number of zero‐count observations in RNA‐seq. A) For each protein‐coding gene in RNA‐seq.A dataset (*n* = 278,^[^ ^133^ ^]^) the mean is calculated by averaging all non‐zero count values, and percentage samples with zero counts are plotted. The insert shows the full range of read counts, and the main plot is zoomed in to the range of 1–10^3^ read counts. The blue box highlights that for many protein‐coding genes, even with relatively low reads, there are no zero counts across most of the range of counts. The red box highlights that for low abundance counts, the range of zero counts ranges substantially, from <20% to close to 100% of clinical samples. B) For the same plot in (A), instead of mean read counts in RNA‐seq, mean HTA 2.0 array signals of the same genes are plotted on *x*‐axis to show the relationship between the gene zero‐count frequency in RNA‐seq and gene abundance detected in the array. As we have noted when comparing the protein‐coding genes detected in the large RNA‐seq datasets (^[^ ^116^ ^]^ Figure 2), a few thousand protein‐coding genes missed by the RNA‐seq studies. Many of the lower abundance genes would not have zero counts in some samples if the cDNA library step reliably reflected the composition of the transcriptome or to a lesser extent, the sequencing was deeper (resulting in more time and costs). Thus, if we plot the abundance value for a protein‐coding gene using the HTA 2.0 data and the zero‐count frequency for that gene from the RNA‐seq analysis, it becomes more evident that many robustly expressed genes show zero counts when they should not (purple box). In contrast, the second group of zero‐count genes is likely a reflection of their low abundance and lack of inclusion in the cDNA library (orange box). Note for the HTA 2.0 array platform, the minimal signal for a genuinely expressed muscle transcript is in the 2.0 to 3.0 log2 signal range. Further details of the code and input data can be found here https://doi.org/10.5281/zenodo.7430956.

Two further conclusions can be reached if we consider the relationship between the number of samples in a study with zero counts (for each gene) and the average expression (excluding the influence of those zero values). First, when we plot the frequency of zero counts (RNA‐seq), against the relative abundance of protein‐coding genes determined by RNA‐seq or array we can see that many genes even with relatively low counts, for example, 6–50 reads, have consistent signals in all samples (blue box in **Figure** **5**A). Second, many protein‐coding genes with >50 counts are detected only in a subset of human muscle tissue (Figure 5A, red box). Some of these may relate to variations in phenotype. However, when we utilize the gene expression abundance values from the array data, we also observe that abundance does not explain “zero counts”, as there are genes with low signal (array) yet have zero counts in 0–100% in the biological replicates (Figure 5B, orange box), as well as robustly expressed (array) genes (Figure 5B, purple box) with zero counts. Together these observations indicate that zero counts do not reliably reflect lack of expression^[^ ^158^, ^159^ ^]^ but reflect other technical deficits in the RNA‐seq process. Heuristics to include genes across fewer than 100% of all samples might help address the question of whether a gene is expressed in a subgroup of patients, yet it then alters the working sample size for data modeling, complicates the definition of “background expression” and the threshold chosen should be gene specific and thus complex to implement.

## 5 The Influence of RNA Processing Protocols

Sample processing steps impact what is discernible by any transcriptomic technology.^[^ ^160^ ^]^ However, certain steps are probably far more influential than others. Ribosomal RNA (rRNA) represents most of the RNA in a clinical sample and will account for >80% of all sequencing reads if not removed (notably, rRNAs are physiologically relevant in the context of aging and growth). Thus, rRNA is usually depleted using hybridization‐based capture approaches^[^ ^161^ ^]^ prior to making the cDNA library for RNA‐seq (leaving behind <20% of the rRNA). Alternatively, oligo‐dT primers can be used to enrich the cDNA library in polyadenylated (poly‐A) transcripts (avoiding rRNA). Libraries prepared with rRNA depletion can better represent the diversity of RNA molecules (e.g., those without poly‐A tails), whereas those using poly‐A enrichment primarily detect protein‐coding genes.^[^ ^161^ ^]^ Depletion protocols for rRNA are in turn sensitive to RNA purification methods.^[^ ^151^ ^]^ Both of these methods introduce bias, for example poly‐A selection approaches fail to capture the expression of repetitive non‐coding RNA elements—which can be important for defining treatment responses.^[^ ^162^ ^]^ These two methods also alter gene quantification among commonly detected genes, resulting in ≈50% of genes demonstrating DE from the two cDNA libraries created from the same RNA sample.^[^ ^162^ ^]^ This result is predictable as many protein‐coding transcripts can be poorly polyadenylated, yet it illustrates that the choice of RNA processing strategy is hugely influential.

As well as the additional costs, time, and variation introduced by the rRNA depletion protocol, cDNA libraries created following rRNA depletion typically require greater depth of sequencing (≈2.5×) to achieve comparable coverage of protein‐coding genes as poly‐A enrichment.^[^ ^163^ ^]^ Further, rRNA is not the only high abundance RNA species, for example, mRNA encoding mitochondrial genes (in highly metabolic tissues) or globin RNA in the case of blood, and the contribution of these genes to total reads is highly variable across samples.^[^ ^133^ ^]^ The result is that there is large variation in the effective depth of coverage per sample, that is, the sequencing depth for most of the transcriptome. Avoiding poly‐A‐based selection strategies may enable greater inclusion of non‐coding RNA in the cDNA library^[^ ^161^ ^]^ however the library remains deficient in lncRNA molecules, and this has led to erroneous conclusions regarding cell‐specific patterns of lncRNA expression.^[^ ^2^, ^54^, ^144^, ^146^ ^]^

Further, variation in RNA preparation is apparent when analyzing formalin‐fixed and paraffin‐embedded (FFPE) samples—a storage choice for many clinical histopathology specimens. RNA derived from FFPE samples suffers from degradation and is poorly suited to poly‐A enrichment protocols. Following rRNA depletion, coverage can be limited, with reports of only ≈20% of sequencing reads mapping to coding regions^[^ ^157^, ^163^ ^]^ while more recently others^[^ ^164^ ^]^ report better success from FFPE (>80% mapping), particularly with large amounts of RNA (1 µg). Often degraded, substantial additional quality control steps are required to remove artifacts from the FFPE RNA‐seq data.^[^ ^165^ ^]^ Array protocols can reliably profile the transcriptome from FFPE samples,^[^ ^34^ ^]^ producing results at least comparable to fresh‐frozen tissue analyzed by RNA‐sequencing.^[^ ^166^ ^]^ Thus, the necessity for RNA selection protocols to remove high abundance RNAs during short‐read RNA‐seq workflows introduces bias and extra time and costs and no current protocol resolves all the identified issues.^[^ ^182^, ^183^, ^184^, ^185^, ^186^, ^187^ ^]^ We recommend that the high‐density array is used with the whole‐transcriptome (WT) reagent kit^[^ ^167^ ^]^ as it does not require RNA selection prior to amplification or use of PCR and is associated with a broad representation of both coding and noncoding RNA.

## 6 The RNA Amplification Step: PCR‐ versus T7‐Based Linear Methods

Each cell expresses a physiologically biased subset of the genome that reflects function. The abundance will also vary, and a cell may express some transcripts at >1000 copies, while many are expressed at <20 copies.^[^ ^168^ ^]^ For most RNA‐seq technologies, quantification of transcripts expressed at these lower levels requires amplification of the input RNA and this is accomplished predominantly using PCR, a method^[^ ^169^ ^]^ that enabled next‐generation sequencing by allowing amplification of adaptor‐ligated cDNA in hours, instead of days. However, not all RNA species are amplified with the same efficiency, and some are lost, while others are selectively amplified. As many mRNAs for signaling proteins or transcription factors are expressed less abundantly than those coding for structural or metabolic proteins, biased amplification impacts on pathway analysis^[^ ^82^ ^]^—where the background of “expressed” genes is ill‐defined^[^ ^79^ ^]^—see below. Furthermore, any biases introduced by the RNA isolation steps can be magnified with each PCR cycle, and such events are not resolvable using informatics.^[^ ^170^ ^]^ Errors introduced before or during PCR amplification are detectable using bar‐coding or unique molecular identifiers (UMI). UMI attempt to identify PCR duplicates (multiple reads amplified from the same cDNA molecule^[^ ^170^ ^]^) and indicate that the most influential factors for PCR duplication events are RNA input and sequencing depth. Interestingly, UMIs are now being used to assemble “synthetic” long reads from standard short‐read sequencing of the fragmented cDNA library.^[^ ^71^ ^]^

In contrast, profiling of the transcriptome using arrays can be done without amplification using biotin‐labelled first‐strand cDNA synthesis.^[^ ^97^ ^]^ More commonly in vitro transcription (IVT), based on Eberwine's work on linear RNA amplification,^[^ ^171^ ^]^ is used instead of PCR and only requires an RNA input of 100 ng.^[^ ^167^ ^]^ IVT involves cDNA synthesis, first primed with an oligonucleotide containing a T7‐phage promoter recognition site.^[^ ^172^ ^]^ Following double‐stranded cDNA production, T7 polymerase is added and directs the synthesis of antisense RNA from the cDNA template, yielding cRNA (also called aRNA—“antisense RNA”). This approach can amplify input RNA one million‐fold after two cycles, only relying on RNA from a single cell.^[^ ^173^ ^]^ The amplified aRNA pool is reported to closely resemble the composition of the original input mRNA population, suggesting that the amplification process has limited bias.^[^ ^173^ ^]^ It also appears that each RNA, especially for lower abundance genes, is more consistent across samples, compared to PCR‐amplified RNA.^[^ ^174^ ^]^

Thus, it is assumed that library strategies produce an amplified DNA library that faithfully reflects the original RNA input. Any major deviations from this assumption have major implications for cDNA library composition and the validity of downstream informatics analyses.^[^ ^175^, ^176^ ^]^ An IVT approach has been adapted for RNA‐seq and was noted to remove several PCR‐related biases and more closely approximate the original sample composition^[^ ^177^ ^]^ but it is not routinely used. The more recent adoption of cost‐effective UMIs and 3’ biased sequencing protocols, reduce the influence of PCR artifacts, but cannot then address alternative exon usage (“splicing”)—something RNA‐seq was supposed to deliver. UMI barcoding is also a common strategy for single‐cell and single nucleus sequencing, where transcript signals are estimated at a gene level.^[^ ^178^ ^]^ Direct RNA sequencing is an alternative and very promising approach for transcript characterization (including RNA editing and allele specific analysis) that remains in development,^[^ ^179^ ^]^ requiring large amounts of starting materials, limited throughput, and high costs.

In short, there are no good methods to account for the preferential amplification of certain mRNA molecules and the dropout of others when PCR amplification is relied on, as is the case for most RNA‐seq protocols. All solutions that might address the fundamental characteristics of either PCR‐based cDNA library or count‐based sequencing also appear to introduce bias.^[^ ^176^ ^]^ The choice of library preparation kit substantially influences study outcomes; thus, without due consideration, many of these factors can explain the lack of replication of detailed findings across laboratories when using RNA‐seq^[^ ^180^ ^]^ or when a comparison is made between RNA and proteomics.^[^ ^57^ ^]^ In our view, the best strategy is to avoid PCR amplification (including any array protocol that uses PCR).

## 7 Data Analysis Steps that Contribute to the Performance Characteristics of the Transcriptome Profiling Technologies

The type of the raw data produced by arrays and sequencing differs fundamentally. While in‐depth discussion of all the implications is beyond the scope of this review, we will explore some of the key differences. Regardless of the method, the raw “signal” must be processed through several steps, including quality control, normalization, quantification/scaling, and summarization of gene expression (ideally at the transcript level). These steps enable statistical comparisons for the same RNA across conditions or relative to a clinical phenotype. RNA‐seq analysis needs to implement a model to estimate how each read relates to the transcriptome, then to “count.” In contrast, Affymetrix array probes are manufactured as a fixed 25‐mer DNA sequence (which needs to be checked for specificity). Data processing of modern array data relies on few options, such that there is a good consensus after ≈17 years of use (1998–2015). Analysis of RNA‐seq data has proven far more challenging and 15 years on (2007 to 2022) there is no consensus; and one is unlikely to be reached that serves all types of data.^[^ ^115^, ^181^, ^182^, ^183^ ^]^ Indeed, major limitations of the most frequently used RNA‐seq computational tools, applied to clinical sample sizes, have only recently been uncovered,^[^ ^153^ ^]^ implying that many “unknown unknowns”^[^ ^184^ ^]^ remain. Arguably more informaticians than ever are working with transcriptomic data; far more than ever worked with array data, which conveys the nature of the challenge ahead for reaching a consensus for the most appropriate informatic solutions for all types of novel transcriptomics.

Before discussing the distinct signals produced by arrays and short‐read RNA‐seq, it is worth briefly commenting on laboratory validation methods. Real‐time qPCR^[^ ^185^ ^]^ is often portrayed as a gold standard validation tool in transcriptomics. The SEQC transcriptomic quality assurance concluded that two qPCR results were no more reliable or quantitative than two sequencing results; presumably because both rely on PCR.^[^ ^122^ ^]^ Real‐time qPCR has no standard for RNA input and lacks widely adhered to laboratory standard conditions. It is also often used without regard to the exact RNA sequence (pre‐configured kits) and can produce a signal from a very minor component of a tissue biopsy (a positive or a negative characteristic depending on how the data is being presented). That real‐time qPCR has been put forward as a “gold standard” tool for validating the results of genome‐wide transcriptomics is not, in our view, logical. Not least because when used, only a few of the “best candidates” from the transcript wide profiling are selected. Real‐time qPCR also relies on housekeeping strategies for quantification, distinct from global methods and often does not measure the same sequence as the global method. Carefully considered real‐time qPCR can be useful for qualitatively confirming differential exon usage, but when used to claim validation of new informatic models, transcriptomic identified candidates should be selected from across the range of false discovery rates (FDR) noted in the study^[^ ^54^ ^]^ to better illustrate the model's true performance.

### 7.1 Processing the Signal from Modern Arrays

Arrays produce a continuous signal, for a given RNA, via hybridization of labelled fragments to short 25‐mer DNA probes dedicated to a single RNA sequence. These probes are combined to estimate the abundance of a transcript, and at the transcriptome level, these continuous signals are approximately normally distributed when plotted on a log_2_ scale. Early in their development the scanners used to process arrays had a limited dynamic range easily leading to signal saturation. In practice, a majority of probe‐sets show a wide dynamic range—at least 7 log_2_ units^[^ ^100^ ^]^ or more. For example, across muscles from 191 sedentary fasted adults,^[^ ^2^ ^]^ the signal for the mitochondrial gene, pyruvate dehydrogenase kinase‐4 (*PDK4*, ENST00000473796) staggeringly varies from 141 to 2800 unlogged units. Interestingly, this heterogeneity might explain why PDK4 is frequently identified as differentially expressed in small human studies.^[^ ^186^ ^]^ Signals from the HTA array probe‐set that detects expression of a contractile protein known to vary greatly across people, Myosin Heavy Chain IIx (*MYH1*, ENST00000226207,^[^ ^187^ ^]^), ranges from 14 to 7660 unlogged units.^[^ ^2^ ^]^ This illustrates, contrary to predominant opinion, that modern arrays possess considerable dynamic range.^[^ ^69^, ^70^ ^]^ The continuous nature of the signal from modern high‐density arrays enables well‐established statistical methods for DE and regression analysis, with limited raw data processing.^[^ ^129^, ^130^ ^]^ The lack of 3’ bias (as probes are designed against all exons and both untranslated regions (UTR) ) and the more quantitative nature of the GC corrected signal should also help when modelling differential exon usage.^[^ ^54^, ^188^ ^]^ On the other hand, the HTA array is not optimal for profiling small RNAs, such as mature miRNAs, as these are shorter than a single probe, and here—with the help of a modified library protocol—sequencing may have some advantages.^[^ ^125^, ^126^ ^]^

Array and RNA‐seq data requires “normalization” to be made more comparable across samples, prior to group based statistical analysis and such methods assume that most of the transcriptome remains unchanged across conditions, such that most data acts as a housekeeping signal.^[^ ^189^, ^190^, ^191^ ^]^ Physiology studies, often using small sample sizes, reporting that 50% of genes are differentially regulated, are for this reason, unlikely to be correctly processed. We have noticed this is not uncommon with RNA‐seq studies and speculate it may also reflect the influence and mishandling of zero counts. Pre‐processing and normalization algorithms for arrays, such as MAS5, PLIER, and dChip, are no longer used and the most common method employed for Affymetrix arrays is robust multi‐array averaging (RMA).^[^ ^192^ ^]^ Packages that implement RMA can to some extent increase low‐level correlative structure across the transcriptome while compressing the dynamic range of the data.^[^ ^193^ ^]^ Iterative rank‐order normalization (IRON) allows the combination of some of the most favorable features from dChip, MAS5, and RMA—yielding some improvements in performance.^[^ ^193^ ^]^ The default implementation of IRON is far more time and data‐storage efficient (implemented in C program via terminal) than RMA (implemented in aroma.affymetrix R package) and is our preferred choice. Using IRON, one can process new samples, without reprocessing all previous samples (something that RMA cannot do). We also prefer IRON as it appears to limit introduction of correlative structure compared with RMA, a factor that can influence network or classification analyses.

Normalization is applied within a study and to a particular array type. Merging raw data across different technologies remains a risky pursuit, despite some enthusiasm,^[^ ^194^ ^]^ and it is not advisable in our view—not least because the probe design varies across arrays, use of a gene level identifier can hide substantial differences in the sequence that is being quantified. Methods developed to remove batch‐related “noise” cannot easily distinguish biological from technical variation in routinely collected samples.^[^ ^195^, ^196^ ^]^ The large number of technical considerations^[^ ^197^ ^]^ and a lack of substantial positive control data (coupled with pathway level validation issues discussed below), means that merging raw data across technologies is not likely to be a valid pursuit. Meta‐analysis of individually processed data is a reasonable alternative.^[^ ^198^ ^]^

There are nonetheless some options prior to applying statistical analysis to data generated by the modern array. For example, using an HTA array with a custom CDF typically produces 100 000 rows of data (probe‐set) summarized at the transcript ID level. In the processed data file, each row represents a transcript (e.g., ENST) but includes recycled data—as an exon can belong to more than one transcript. It is not easy to know a priori which ENST is the more representative transcript for your experiment, and many cells express more than one variant.^[^ ^78^, ^199^ ^]^ It is possible to select one expressed ENST per gene (resulting in ≈30 000 rows of data (genes) for a human tissue profile) based on which shows the greatest signal or greatest variation, or some heuristic combination, across your samples or between groups. This remains a stage in array data processing that could be optimized using machine learning. Overall, the narrow suite of analytical options for processing modern arrays ensures that it is much easier to track analysis from the raw data, promoting research transparency, and reproducibility. Thereafter, DE analysis can be carried out using simple ANOVA models combined with correction for multiple testing or utilize methods such as Significance analysis of microarray data (SAMR) that models a false discovery rate.^[^ ^129^, ^130^ ^]^ Modest changes in array analysis software have tended to only lead to subtle changes in the outcome of any analysis—except for the issue of accurately defining the “background” reference for pathway or ontology analysis (see below).

### 7.2 Processing the Signal from Short‐Read RNA‐seq

Computational methods for adequately processing of RNA‐seq data are still evolving, and the optimal choice may be experiment specific.^[^ ^115^ ^]^ Processing raw sequencing data is more complex than for array data, and there are hundreds of potential combinations of methods,^[^ ^115^ ^]^ with dozens in common use.^[^ ^70^ ^]^ A recent comparison of 278 different options, including mapping, quantification, and normalization steps, found that the pipeline chosen profoundly impacted on gene expression analysis.^[^ ^200^ ^]^ Others report that the number of DE genes substantially varies depending on the processing method, from 208 to 9500 DE^[^ ^201^ ^]^—with the later challenging the validity of the normalization or modelling of counts. Other fields have recognized limitations of transforming counts to enable statistical analysis.^[^ ^158^ ^]^ This is particularly problematic in the case of RNA‐seq, where a zero count may reflect a laboratory failure and not the biology of the sample, or failure to sequencing to a sufficient depth (or variation introduced by “effective” depth).

The first step maps short reads to a reference annotation of the genome, a process that can be non‐trivial.^[^ ^108^, ^202^, ^203^ ^]^ The numerous options introduce bias, yet normalization, and DE algorithms are thought to contribute most to the variability between analyses or across studies.^[^ ^200^, ^201^ ^]^ Traditional alignment tools are slow, and more recent methods, that is, Salmon and Kallisto^[^ ^202^, ^204^ ^]^) were introduced to save time and estimate which transcript a read belong to, using graph theory. Salmon adjusts for nucleotide bias, impacting on subsequent statistical analyses^[^ ^204^ ^]^ to debatable extent (https://www.liorpachter.wordpress.com/tag/salmon/). While very informative, Pachter's commentary does not dwell on the 2–4% difference between these two near‐identical methods, a difference that would yield thousands of distinctly quantified transcripts; more than a typical biology‐driven DE signature. In the Salmon paper, >1000 regulated transcripts were found from the simulation analysis when there should be none.^[^ ^204^ ^]^

Results from traditional alignment tools and pseudo‐alignment tools^[^ ^202^, ^204^ ^]^ differ for lower count genes,^[^ ^205^ ^]^ that is, a majority of the rows of “data” in an RNA‐seq study (Figure 5 and see ref. [151]). Further, reads are typically assigned to a gene (and not a transcript) and this obviates the production of information on alternative splicing. Indeed, Stark et al. recently illustrated several additional methodological weaknesses in short‐read RNA‐seq informatics pipelines,^[^ ^70^ ^]^ including that use of “fast” tools that introduce variation^[^ ^205^ ^]^ on how multi‐mapped reads are assigned to the estimate of gene expression (as a source of bias,^[^ ^206^ ^]^). One promising DE strategy, to address the uncertainty regarding which transcript to assign a read too, is to calculate an aggregated p‐value from analysis of each possible option.^[^ ^207^ ^]^ This strategy has also been applied to ontology analysis, but this may be far more challenging to interpret (see below). Further refinement, aggregating DE analysis across exons has also been explored.^[^ ^208^ ^]^

**Figure 6:**
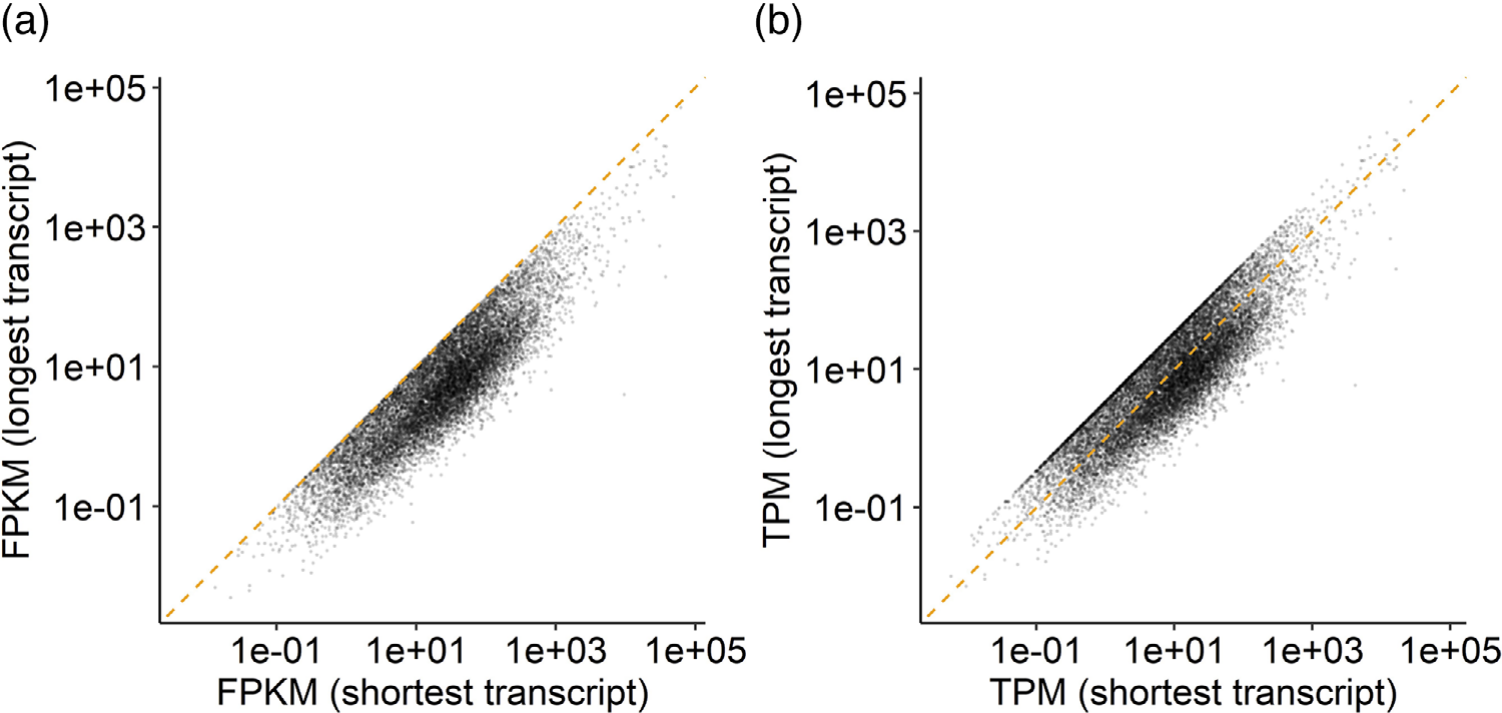
Adjusting RNA‐seq counts by different transcript lengths. To illustrate the issue of scaling to gene length, we plot the same data normalized using two different methods that scale reads to gene size and are supposed to be interchangeable. A) FPKM method is used, and the counts were scaled to either the longest or shortest possible example for each gene. B) TPM method is used, and the counts were scaled to either the longest or shortest possible example for each gene. Further details of the code and input data can be found here https://doi.org/10.5281/zenodo.7430956.

Typically, once reads are assigned to a specific part of the genome, counts can be adjusted to the total number of mapped reads in a sequencing run, for example, counts per million (CPM). At this point, counts are still not comparable between “genes” as counts require adjustment for GC content^[^ ^118^ ^]^ and length related bias. Fragments per million reads mapped (FPKM) and the transcripts per million (TPM) are attempts to scale counts data within a sample^[^ ^108^ ^]^ or if multiplexed using barcodes, within a sequencing lane. Note that multiplexed sequencing assumes that each sample contributes equally to the library prior to sequencing. Introduction of these types of transformations led to expectations that count values would be quantitative, and comparable across labs, or sequencing runs.^[^ ^134^, ^209^ ^]^ FPKM, aims to correct counts per gene by length (“counts per kilo base”) and library size (total counts in million units) to scale data and allow comparison within sample runs. It does not consider the idea of what we call “effective” sequencing depth (depth after considering variable coverage because of extremely high abundance transcripts). TPM is a related transformation, adjusting the “counts per kilo base” for the total “counts per kilo base” of each sample (million units)^[^ ^209^ ^]^ yet as we illustrate here (**Figure** **6**), the impact of adjustment is not consistent.

A read can be legitimately assigned to more than one transcript from the same gene, and thus it is unknown which transcript “length” to correct for. We illustrate the influence of transcript length by plotting transformed count data (using FPKM and TPM), correcting to the longest and shortest transcripts for each gene. FPKM is systematically influenced by length, and this is a problem as the transcript used may shift between the conditions (Figure 6A). The influence of changing transcript length on TPM values is inconsistent (Figure 6B) because the choice affects both the nominator (“counts per kilo base”) and the denominator (total “counts per kilo base” in each sample). The bias introduced by attempting to scale count data by gene length impacts on downstream statistical models.^[^ ^209^ ^]^ Adjusting by the wrong transcript length results in DE under conditions when differential splicing has occurred or as articulated by Stark et al.;^[^ ^70^ ^]^ “if the main isoform in one condition is half the length of the main isoform expressed in the second condition (but expressed at twice the “level”) it would look as if the gene was not regulated”.

Even after conversion of a read into a “scaled” count, the data are not necessarily “normalized” in the traditional sense applied to arrays or protein blots.^[^ ^210^ ^]^ Global normalization requires a general consistency of RNA expression to be true,^[^ ^211^ ^]^ but in projects sequencing libraries from clinical tissue samples, reads for very high abundance genes can vary dramatically across samples, greatly altering the fraction of total reads they make up. This means that the effective sequencing depth across samples—for most genes—is not well scaled by total aligned reads. Systematic flaws in scaling strategies could lead to a high proportion of genes being called DE. Recently, graph‐based strategies have explored normalization methods that may not be so dependent on these traditional assumptions.^[^ ^212^ ^]^ However, these methods, benchmarked by DE metrics, may not be valid for all types of analyses, while attempts to fit the count‐based data to a distribution model, appropriate for DE methods, are less accurate and control FDR less well than desirable.^[^ ^210^ ^]^ Stark et al. highlight that iterative evaluation and selection of a normalization procedure may be critical to discover the validity of your pipeline, but final choices must not be based on the outcome that fits best with the original hypothesis.^[^ ^70^ ^]^ Indeed, to avoid such a scenario, the inclusion of two or more independently produced clinical data sets should be incorporated in a single publication, to validate any pipeline choices.^[^ ^37^, ^213^, ^214^ ^]^

Thus, if the analysis of an RNA‐seq data set is to be done thoroughly, many parallel data processing options should be considered,^[^ ^70^ ^]^ but this is almost never done due to a lack of specialist expertise or perhaps a rush to publish. The increasing number of models, especially those evaluated using simulated data, may never solve the inherent limitations of the RNA‐seq laboratory steps.^[^ ^211^, ^215^ ^]^ It has taken nearly a decade to identify limitations^[^ ^153^ ^]^ of the most utilized RNA‐seq analysis tools (DESeq2 and edgeR)—despite over 65 000 citations—and it may take years for updated solutions to be fully explored. To complicate matters further, RNA‐seq software packages are frequently updated, and the use of different versions affects the results obtained.^[^ ^216^ ^]^ For example, the choice of pipeline to analyze tumor samples impacts on survival predictions.^[^ ^200^ ^]^ Lack of reporting software versions and lack of clarity over optional settings for your study, will drive a lack of reproducibility.^[^ ^116^ ^]^ Thus, specific recommendations for processing RNA‐seq data are not easily made—and this would normally limit the utility of the technology for translational or stratified medicine. Nevertheless, RNA‐seq has become the dominant technology and there is now an urgent and ethical prerogative that these limitations are more widely discussed, not least because alternatives methods exist.

### 7.3 Implications of Bias for Pathway Analysis and Other Downstream Analyses

Identifying DE genes to then model which pathways are regulated, represents a very common type of OMIC analysis. Most methods used for DE analysis of RNA‐seq data, typically involves small sample sizes, and these same methods do not appear to control the FDR adequately in larger clinical sample cohorts^[^ ^153^ ^]^ (how they adequately control FDR in a sample size of three biological replicates is also unclear). Methods applied to array data to calculate DE are well established and, in our experience, appear robust across independent data sets.^[^ ^54^, ^129^, ^130^, ^217^, ^218^ ^]^ Linear modelling or logistic regression can be applied to array data^[^ ^28^ ^]^ and reliably identify genes correlated with a clinical status, even across a variety of types of arrays.^[^ ^2^, ^26^, ^35^ ^]^ Unless filtered to model genes only expressed in all samples, regression modeling of rows of RNA‐seq data would encounter lots of missing data,^[^ ^120^ ^]^ with the actual sample size (per rows or column) impractical to define. For quantitative network analysis, modeling can utilize databases (e.g., protein‐protein interaction data) or take a direct data‐driven approach.^[^ ^219^ ^]^ The basic principles^[^ ^220^ ^]^ of data‐driven quantitative network analysis apply to all OMIC methods, whereby hundreds (or even thousands) of independent samples are required to produce stable network results.^[^ ^220^, ^221^ ^]^ Most researchers do not adhere to such criteria, instead favoring methods that are simple to implement yet do not provide reliable estimates of network stability or adjust for multiple correlations.^[^ ^219^ ^]^ Database driven pathway or gene‐ontology analysis^[^ ^222^, ^223^, ^224^ ^]^ is used in many transcriptomic projects and increasingly to summarize the conclusions of multi‐omic analyses (where their validity is especially unclear)—and often relies on web‐based tools^[^ ^222^, ^223^, ^224^, ^225^, ^226^, ^227^ ^]^ which can be difficult to replicate.

Pathway‐based analysis represents an additional “statistical” hurdle beyond DE analysis—one that should confirm that a list of DE genes reflects the biology of the experiment and not one driven by noise or chance.^[^ ^82^ ^]^ Pathway analysis can start with a list of DE genes, identified by choosing an FDR threshold, that is, an approximation of what is regulated. These adjusted p‐values (FDR) do not confirm that the driver for DE is biological—it could still be driven by bias relating to RNA handling, cDNA library production or sample processing order. Most transcriptomic studies include a table or plot of the enriched pathways as a major general summary of the study. A recent survey identified that >80% of published pathway analyses have serious problems,^[^ ^79^ ^]^ quantifying our earlier concerns.^[^ ^82^ ^]^ In fact, the assumptions for calculating valid p‐values for enrichment analyses are also known to be violated (anti‐conservative) to some degree.^[^ ^81^ ^]^ In general, in our experience, pathway FDR values in the 1 × 10^−2^ to 1 × 10^−5^ range probably do not reflect genuine differences, but rather defects in the informatics procedures and biases in the laboratory method.

**Figure 7:**
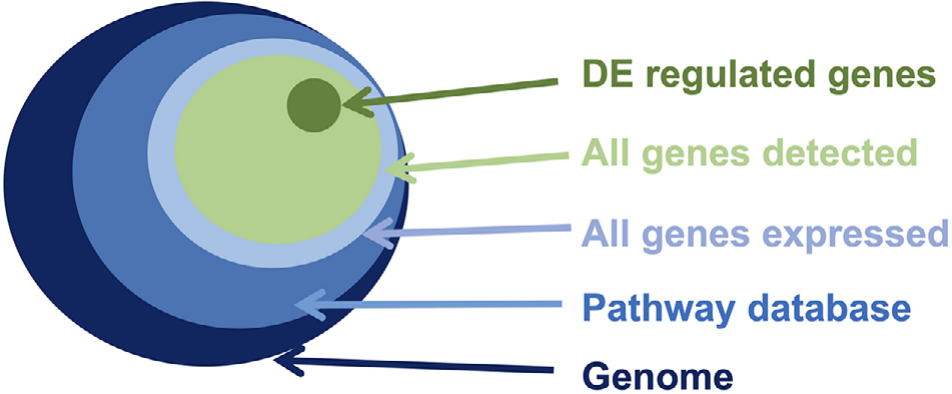
The categories of gene sets applied to functional enrichment analysis. The correct comparison for any gene ontology enrichment analysis is to compare the set of DE genes (dark green) with the set of genes detected (light green) as the background (light blue represents the perfect technology measuring the correct background). Using any other gene list as background will result in false statistical enrichment categories (reflecting tissue and technology biases) and will obscure real events. Further, if comparing two treatments (e.g., drugs) then using the incorrect background can remove valid GO categories from both sets of analysis and thus obscure any differential influence of each treatment on the pathway biology.

It is simple to carry out a “less biased” pathway analysis, but the consequences are usually loss of any significant results because, in our experience, many results are driven by inappropriately defined background^[^ ^79^ ^]^ transcriptome. This is especially true when you model a targeted proteomics data set and contrast that data with the ontology database or genome, rather than the narrow set of proteins measured. To calculate if a pre‐defined pathway (ontology) is enriched in a DE gene list, the list must be compared with all genes that were possible to measure.^[^ ^82^ ^]^ The list should not be compared with the entire content of a database or the genome (**Figure** **7**) as this largely reports on differences between the biology of your sampled tissue and the database.^[^ ^79^ ^]^ Failure to use the appropriate background generates significant FDR values irrespective of whether a real DE gene list or one reflecting an artefact is used.^[^ ^79^, ^82^, ^228^ ^]^ While it can be reasonably clear which genes were measured in an array experiment (Figure 7, Pale Green Circle), as we have illustrated above, this is not the case for short‐read RNA‐seq—where for the same tissue, the background protein coding transcriptome varied dramatically. In RNA‐seq data consistently expressed gene can be identified using thresholds of a minimum of 5, 8, or 16 raw counts per gene^[^ ^122^ ^]^ and some metric for the number of biological replicates that should exceed this threshold. Alternatively, a minimum of 1 count per million reads (5–100) has been used, yet it is understood that some minimal level of signal may reflect genomic DNA and other artefacts.^[^ ^70^, ^176^, ^221^, ^229^, ^230^ ^]^ When data quality is poor, authors often choose thresholds of <1 count on average across samples to define the detected genes and include genes that are not expressed in many of the samples.^[^ ^120^ ^]^ This approach not only influences the DE analysis, but it renders the definition of the background transcriptome a moving target.

**Figure 8:**
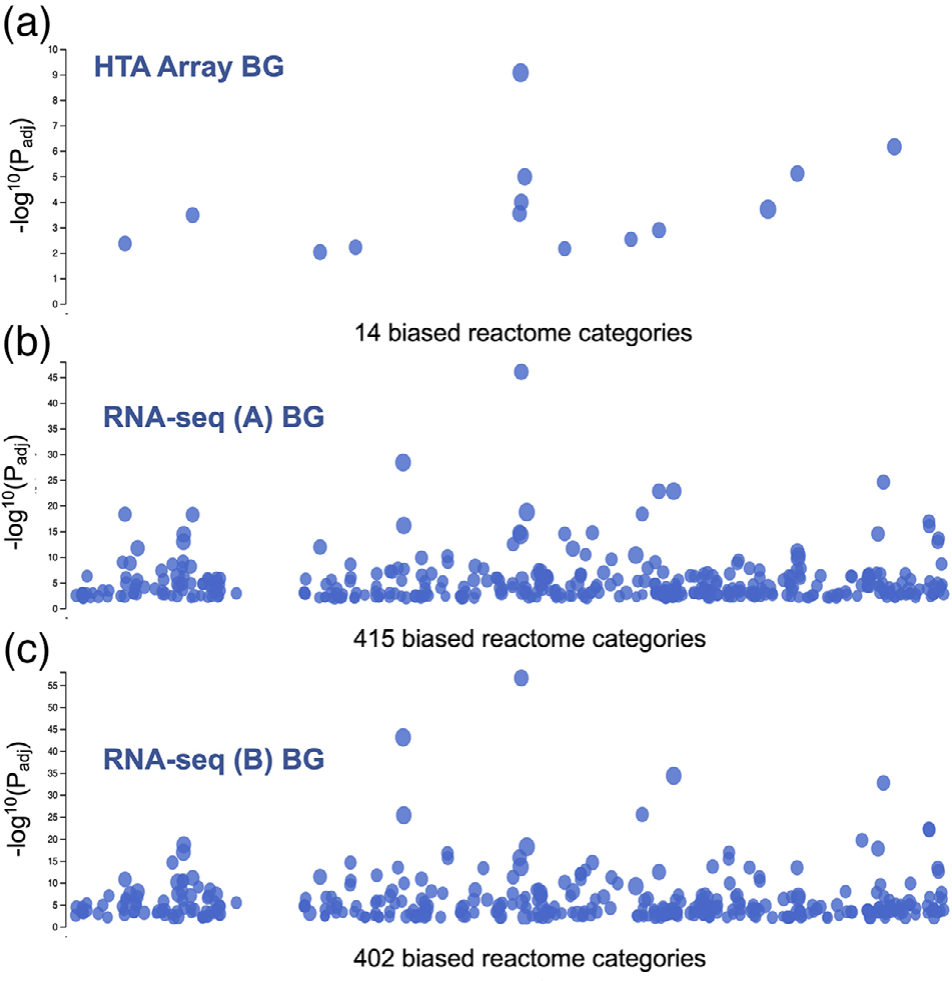
Comparison of pathway‐level bias in muscle background transcriptomes derived from RNA‐seq and the HTA array. In each case, the genes detected, as described in Figure 1, were contrasted with the Reactome database using g:Profiler and default settings. The significant pathways represent bias of muscle tissue versus the genome as represented by the Reactome database, and bias introduced by the laboratory method. A). HTA array transcriptome, with a total of 25197 gene IDs mapped to the database, obtained 14 enriched pathways, many of which reflected aspects of muscle biology. B) RNA‐seq data set A, with 15139 gene IDs mapped to the database, obtained 415 enriched pathways. C) RNA‐seq data set B, with 13841 gene IDs map to the database, obtained 402 enriched pathways. P values are BH adjusted ‐log_10_ values. The constitutively expressed “background” transcriptome as measured by RNA‐seq displayed bias for an enormous range of generic processes, many of which appear methodological rather than biological in origin.

In the example provided for RNA‐seq performance across clinical studies (Figure 1), we illustrate that each yields a distinct detectable background transcriptome for human muscle. Plotting the results of each different background list with the consistent and default contents of the pathway database, readily exposes the pathway level bias in these “backgrounds” (**Figure** **8**). In this example we used g:Profiler and the Reactome database^[^ ^224^ ^]^ and three RNA‐seq backgrounds and report that each lists exhibit different biases, only some of which reflects the specialized biology of muscle tissue. The HTA array “detectable background” also had some bias, but in this case for only a few biology‐driven metabolic and contractile‐related pathways, while the rest had moderate adjusted p‐values (Figure 8A) which can arguably be ignored by using a more conservative pathway enrichment threshold. In contrast, for two RNA‐seq data sets, >400 significant categories are noted (Figure 8B,C), many with 1 × 10^−6^ adjusted p‐values or better. Sampling sets of a few hundred genes at random, to mimic a DE list from this data, would yield some of these significant pathways each time. Thus, even when a study is underpowered, and the validity of the DE analysis is doubtful, “highly significant” pathways will still be “discovered”. This problem is not removed simply by larger sample sizes^[^ ^231^ ^]^ or deeper sequencing because it also reflects what we defined as “technology” bias^[^ ^82^ ^]^—factors inherent in the laboratory steps.

**Figure 9:**
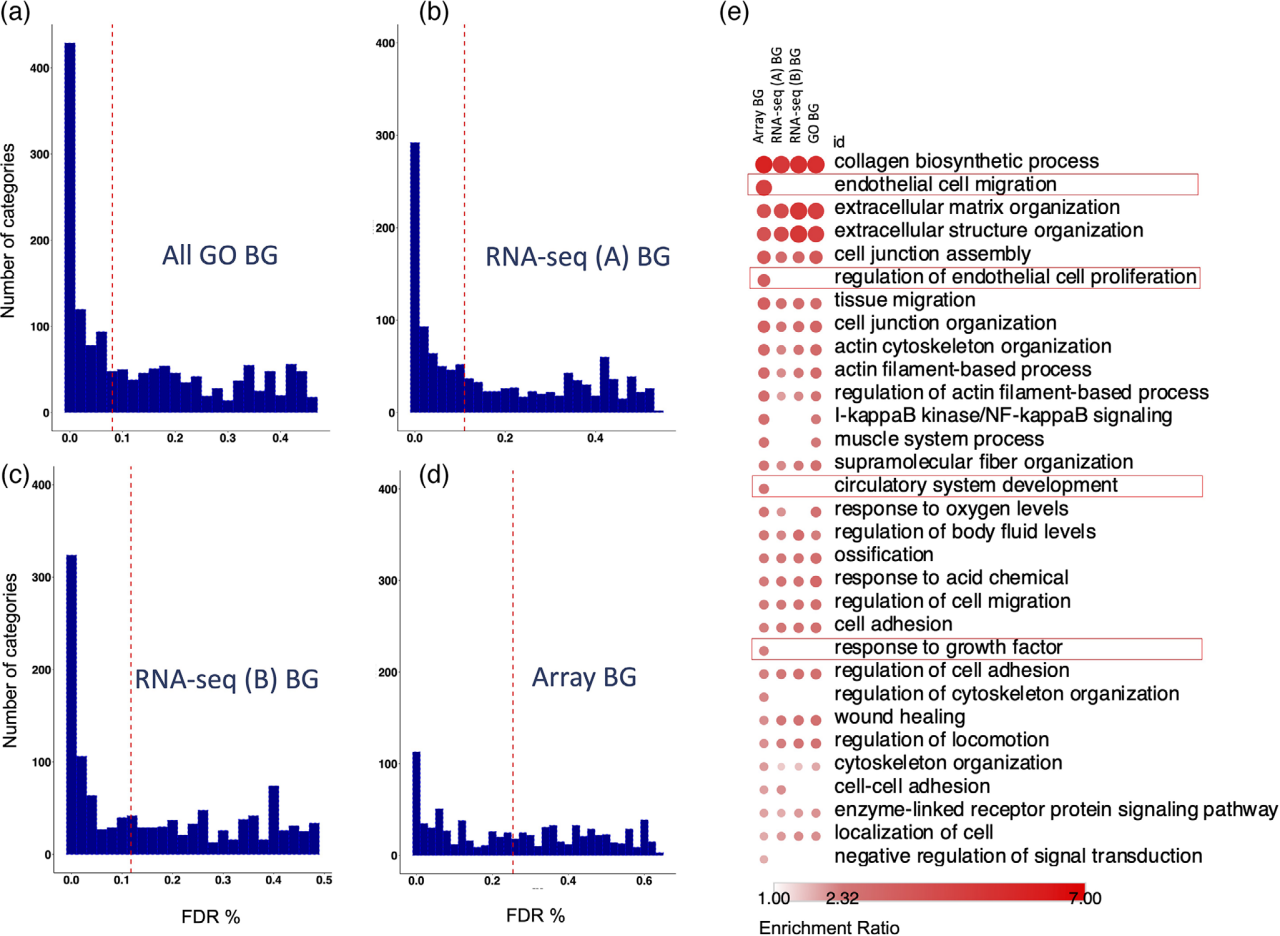
Functional enrichment pathway analysis using four distinct background transcriptomes. The background genes detected are the protein‐coding genes from Figure 1 or the DAVID database (April 2022). The 729 DE genes were contrasted with each background option using DAVID, with a minimum of 3 genes per category and 10000 permutations for estimating the FDR. The red line represents the median FDR across the analysis, and values are plotted for all ontology categories in the analysis (A) uses the entire DAVID database as background, (B) uses the RNA‐seq data set A as background, that is, 12190 protein‐coding genes (C) uses the RNA‐seq data set B as background, that is, 9389 protein‐coding genes and (D) uses the HTA array muscle transcriptome as background, that is, 18 605 protein‐coding gene IDs (SD of >1 filter). The average FDR is a greater value (“less significant”) when using the least biased background, as it better reflects all of the genes that could have been included in the 729 DE gene list. All other backgrounds introduce bias and yield false GO categories. E) Use of the wrong background removes key modulated pathways. The 729 DE genes from an established clinical model^[^ ^8^, ^31^, ^39^, ^232^, ^233^ ^]^ were processed with each background option in Figure 9A‐D using DAVID. Pathway categories with the range of 3–10000 genes per category were used. To remove redundancy (in terms of GO terms) those results were processed using REVIGO (Resnik, least redundancy) before a heatmap of the results was plotted using Morpheus (https://www.clue.io/morpheus). The fold enrichment values are used to color the categories—with a blank value meaning that the category was not significant (FDR >5%). The boxes highlight key processes that were only detected using the background produced using the HTA array.

Additional problems are revealed when a single list of DE genes is compared with the different backgrounds generated from muscle tissue. In our example, a 729 DE gene list was obtained from a clinical signature of muscle tissue adapting to exercise,^[^ ^31^ ^]^ and these genes are involved with extracellular matrix remodeling, angiogenesis, and metabolic adaptation^[^ ^8^, ^31^, ^39^, ^232^, ^233^ ^]^—well validated processes. The biological process (BP) ontology category in DAVID^[^ ^222^ ^]^ was used, with the entire BP ontology database (obviously incorrect as it contains genes that are not expressed in the experiment) and three muscle background transcriptomes used to analyse the 729 DE list (Figure 8). There were >450 or >300 significant BP ontologies (FDR <5%) enriched when using the DAVID database background or an RNA‐seq background, respectively (**Figure** **9**A,C), of which 162 are unique to the database background, and 59 are unique to the RNA‐seq background. Use of the array‐based muscle expressed background resulted in ≈50% fewer BP categories, with 178 non‐redundant GO BP processes being significant (Figure 9D) with a median FDR of 25%. Alarmingly, using the DAVID GO BP background^[^ ^79^ ^]^—not so unusual to observe—yields a median FDR of between 5% and 10% for all categories! Many of the GO BP ontologies deemed significant using these inappropriate backgrounds, tended to have modest fold enrichment ratios and this metric can be used as an additional way to filter out bias driven enrichment results. Notably, the common complaint that pathway results are often generic in nature may reflect the widespread misuse of the method.^[^ ^79^, ^82^ ^]^

Use of an inappropriate background does not just yield false‐positive data, it can also lead to loss of information. Methods such as TopGO or REVIGO can be used to prune the redundant nature of the GO results produced by DAVID,^[^ ^28^, ^223^ ^]^ identifying sub‐categories responsible for driving the enrichment statistics. Applying this approach to the data in Figure 9A–D, resulted in the array background having 68 categories with an FDR <5%, while the RNA‐seq background produces 143 categories. Notably, the use of the RNA‐seq background resulted in several categories related to endothelial remodeling being missed (Figure 9E)—a core physiological processes during muscle remodeling,^[^ ^8^, ^31^, ^39^, ^232^, ^233^ ^]^ reflecting angiogenesis. In contrast, when we utilized an “age gene expression signature”—one enriched in mitochondrial genes^[^ ^30^ ^]^—and repeated the process used for Figure 9E, no enrichment in mitochondrial pathways was noted when using the RNA‐seq background. This may reflect bias in the muscle RNA‐seq data, reflecting greater counts derived from high abundance mitochondrial genes,^[^ ^133^ ^]^ yet clearly it represents a flawed pathway result. In our experience, background bias usually introduces false positive GO results, but as we have illustrated in some cases real observations, reflecting important but subtle gene expression programs, will be missed.

## 8 Modeling Alternative Splicing with Bulk Transcriptomics

Alternative RNA splicing (AS, or alternative exon usage, AEU) is a central determinant of the complexity of the proteome, occurring with >90% of multi‐exon genes.^[^ ^54^, ^144^, ^199^, ^234^, ^235^ ^]^ Accurate modeling of AS/AEU provides information beyond transcript abundance, allowing transcriptomics to capture an even greater proportion of the biological variance in a clinical dataset. Currently, long‐read sequencing and advanced profiling strategies^[^ ^127^, ^179^, ^236^ ^]^ are used to establish if isoform‐specific transcripts are produced or not. Short‐read RNA‐seq was proposed as a method^[^ ^69^, ^70^ ^]^ for routine quantification of AS/AEU events. As exons are shared between isoforms of a gene, mapping RNA‐seq reads to specific transcripts is challenging, and the problem scales with the number of shared exons.^[^ ^234^, ^237^, ^238^, ^239^, ^240^ ^]^ Established methods are mostly suited to detect extreme changes—such as those that occur in cancer or between cell types—rather than during physiological modulation of transcript isoforms, or with less severe changes in clinical disease status. Many reads that are exon‐spanning are also challenging to assign to specific isoforms as they can be shared across several isoforms.^[^ ^70^ ^]^ Clearly, any method—like many newer RNA‐seq protocols—that relies on a cDNA method biased toward the 3’ end of the transcript, cannot study AS/AEU. Recent attempts using iterative models, to assign reads to specific transcripts report improved performance over Cufflinks, SLIDE and StringTie—but still report precision and recall performance at <70% and 40%, respectively.^[^ ^241^ ^]^ This performance may be improved using long‐read technologies. Ongoing attempts to develop laboratory methods, segregating transcripts by length prior to processing and sequencing, partly serves to illustrate the wide acceptance that existing methods to model transcript‐level data need improved.^[^ ^242^ ^]^

Both the HTA 2.0 and the updated version, the Clariom D array, contain probes designed to span individual exons (“junction probes”) in known transcripts, and these were intended to improve the detection of AS of cassette exons.^[^ ^98^ ^]^ There are very few methods for studying AS/AEU using high‐density arrays,^[^ ^243^ ^]^ with only one making direct use of these exon–exon junction spanning probes,^[^ ^244^ ^]^ and one which has subsequently been adapted for RNA‐seq.^[^ ^245^ ^]^ We developed a pipeline to model AEU called iGEMS,^[^ ^54^ ^]^ building on a statistical model from Robinson et al.^[^ ^246^ ^]^ iGEMS aimed to reduce false‐positive results and reliance on laborious visual inspection. Studies of AS/AEU in cells with large treatment effects may indicate a method is working, but it does not establish that the method will be generally applicable.^[^ ^54^ ^]^ Given that many algorithms formulate AEU as an “outlier detection problem”, optimizing the removal of noise, e.g., poorly performing probes prior to signal summarization may also improve existing methods. Thus, in general, AS/AEU modeling of short‐read RNA‐seq and modern array data is challenging and often relies on multiple strategies, including laborious visual inspection of results.^[^ ^54^ ^]^

To illustrate the performance of splicing analysis using RNA‐seq versus the HTA array, consider the 2015 Science article, where RNA‐seq was used to profile multiple post‐mortem tissues.^[^ ^144^ ^]^ This project (GTEx) reported 23–516 AEU events across two‐way comparisons^[^ ^144^ ^]^ including 370 AEU events between adipose and muscle tissue. A major conclusion was that tissue specificity was determined primarily by differences in gene expression abundance rather than through differences in AS/AEU. At the same time, using the HTA 2.0 array^[^ ^54^ ^]^ we identified >1500 AEU events between adipose and muscle (with some independent validation using exon specific qPCR). The splicing ratio statistic used by the GTEx project has high uncertainty at lower count thresholds^[^ ^247^ ^]^ and we noted that the AEU events they reported^[^ ^144^ ^]^ were only among abundant genes and biased for events related to cassette exons. More recently, the muscle FUSION project studied AS/AEU using RNA‐seq, reporting one isoform relevant to metabolic status. This analysis used a simple ratio method to detect AS/AEE and the event was not replicated in a comparable array analysis.^[^ ^2^ ^]^

While there are several explanations for limited AEU detection using short‐read RNA‐seq, including those mentioned above, Xu et al. identified that, at a comparable sequencing depth, variability in expression is high for features with fewer than 20 mapped reads, which included ≈60% of the exons quantified. Evaluation of ten commonly used tools for differential splicing analysis using RNA‐seq, found minimal overlap in the number of genes detected, ranging from 0 to more than 14 000, depending on the method used.^[^ ^248^ ^]^ This suggest that short‐read RNA‐seq has limited utility for large‐scale discovery of AS/AEU, and there is no evidence that it offers better resolution than modern arrays.^[^ ^54^ ^]^ While modeling AS/AEU using arrays provides many candidates, the data will be incomplete and direct long‐read RNA profiling technologies offer a clear advantage.^[^ ^71^, ^179^, ^249^ ^]^ Whether the latter offer reproducible quantification suitable for large‐scale clinical projects remains unknown. There are still opportunities to improve available methods for studying AS/AEU using modern arrays. For example, no method uses the exon‐exon junction spanning probes while properly adjusting the FDR for gene length.

## 9 General Conclusions

Reductions in cost coupled with a number of deep‐rooted misconceptions led to 2nd generation short‐read RNA‐seq becoming the method of choice for most bulk transcriptomics, replacing the microarray. While efforts are being made to reduce costs and provide greater throughput for RNA‐seq these do not address fundamental limitations (e.g., bias in the cDNA library or the nature of count‐based data). Indeed, some modification may make matters worse. Fundamental characteristics of the RNA‐seq methodology, can be shown to compromise the validity of downstream statistical analyses, where numerous biases rather than biology drive significant results. Notably, cost‐effective routine RNA‐seq analyses, using reference databases, do not discover novel transcripts, nor many gene‐splicing events. RNA‐seq also generates huge data files (packed with millions of estimated counts of a few abundant genes), representing an incomplete transcriptomic record of a clinical sample and significant long‐term storage costs. Equally we recognize that 3rd generation direct RNA sequencing technologies are powerful tools for building transcriptome databases, studying allele specific transcription and characterizing their post‐transcriptional modifications. These newer methods do not however appear suitable yet to generate cost‐effective quantitative transcriptomes for large translational medicine projects. Studies contrasting the global transcriptome and proteomics need to reflect on the relative count nature of RNA‐seq data, and better integrate the temporal nature of transcription, translation, and proteostasis. Finally, many genomic databases use RNA‐seq data to define the landscape of the human transcriptome^[^ ^144^ ^]^ and this confounds the literature with inaccurate claims regarding the anatomy of gene expression.^[^ ^250^ ^]^ We strongly advise against using any RNA‐seq reference, commonly linked within genomic browsers, as a view on whether a gene is expressed or not in human tissue, particularly GTEx (which is further confounded by disease, postmortem delay and drug treatment). Together, we resist concluding that short‐read RNA‐seq and arrays are “complementary technologies” and close instead by stating that high‐density modern arrays are a more robust and cost‐effective option for many types of studies, especially when aiming to profile long noncoding RNAs.

## Conflict of Interest

The authors declare no conflict of interest.

## Author Contributions

T.S. and H.H.C. contributed equally to this work. T.S., H.H.C., and J.A.T. drafted the manuscript and completed the literature review. H.H.C. and J.A.T. performed bioinformatics analysis on previously published datasets. All authors critiqued and edited the manuscript.
